# Influence of the industrial pollutant on water quality, radioactivity levels, and biological communities in Ismailia Canal, Nile River, Egypt

**DOI:** 10.1007/s11356-024-32672-9

**Published:** 2024-03-08

**Authors:** Noha Imam, Amr S. El-Shamy, Ghada S. Abdelaziz, Dalia M. Belal

**Affiliations:** 1 Physics and Geology Lab., Freshwater and Lakes Division, National Institute of Oceanography and Fisheries, 101 Kaser El Aini Street, Cairo, 11516 Egypt; 2 Chemistry Lab., Freshwater and Lakes Division, National Institute of Oceanography and Fisheries, Cairo, Egypt; 3 Hydrobiology Lab., Freshwater and Lakes Division, National Institute of Oceanography and Fisheries, Cairo, Egypt

**Keywords:** Abu Za’baal industrial zone, Ecological status, Attached diatoms, Radionuclides, Environmental hazards

## Abstract

In the twenty-first century, numerous forms of pollution have adversely impacted freshwater and the entire aquatic ecosystem. The higher population density in urban areas also contributes to increased releases of substances and thermal contaminants, significantly stressing the ecosystem of industrial companies. This study aimed to assess the potential pressure of industrial and municipal activities on water quality, radioactivity levels, and biological diversity, focusing on the consequences of radionuclides on periphytic diatom communities. Furthermore, the environmental impact of pollutants will be evaluated to monitor the ecological condition of the Ismailia Canal. Chemical analyses employed various instruments and methods to identify and quantify matter, with radionuclide elements measured by gamma spectrometry and diatoms counted and identified by inverted microscopy. Our results revealed that the canal was classified as excellent for irrigation, aquatic life, and drinking water based on FAO, CCME, and EWQS water quality indices, with high nutrient levels at Abu Za’baal fertilizer company. The activity concentration of ^226^Ra-series, ^232^Th-series, and ^40^K in the water and sediment samples for two seasons was within the guideline values, except for a few stations in the zone [B] (the industrial zone). Fertilizer samples (raw material) showed a high value of the ^226^Ra-series activity. Diatom community structure significantly varied across the different canal locations regarding the presence or absence of industrial activities, with no discernible variations between the study seasons. A specific variety of algal species was found to be predominant at the highest radioactive sites. Redundancy analysis (RDA) showed a significant correlation between parameters (pH, Na, TDS, PO_4_, SO_4_, SiO_2_, K, and CO_3_), radionuclides, environmental conditions, and the composition of the diatom community, especially in the area affected by industrial discharges. Moreover, the radiological hazard index in water and sediment remained below the maximum for two seasons. This research provides valuable data and information for communities and decision-makers, suggesting the strategic use of phycoremediation as a water biotreatment process to protect the valuable economic resources of the Ismailia Canal.

## Introduction

The majority of industrial cities around the globe struggle with degraded water quality caused by the influx of contaminants. Industrial activities contribute substantial amounts of contaminants, such as those in non-nuclear industries, including fertilizer production, coal power plants, petroleum and natural gas extraction facilities, and ceramics manufacturing (Brigden et al. 2002). The uncontrolled discharge of industrial wastewater into the aquatic system has significantly impacted the water quality and aquatic life (Begum et al. 2008). Assessing physical, biological, and chemical properties is crucial for evaluating the quality of the water class and its designated uses, including drinking, agricultural activities, and industrial purposes (Abdel-Satar et al. 2017). Industrial wastes usually comprise high amounts of dissolved and suspended substances, heavy metals, and hazardous elements (such as U, Th, and Ra) that may influence the ecology of aquatic organisms, interrupting the entire system and posing direct or indirect threats to human health (Pappa et al. 2016). Additionally, they affect the biodiversity of aquatic biological communities, such as the richness and evenness of species (Li et al. 2022). Environmental stresses may result in declines in diversity or changes in the abundance of aquatic biological communities, while biodiversity metrics assist in describing the numbers and distributions of species (Li et al. 2022).

Environmental pollution is undoubtedly one of the main problems that society faces today. Periodic water quality surveillance in the aquatic ecosystem is essential to assess ecological health and suitability for various purposes, such as agriculture, industry, and domestic (Poonam et al. 2013). For instance, raw materials for producing fertilizers include considerable amounts of natural radionuclides. The manufacturing process of these materials may expose staff members and nearby residents to radiation levels significantly higher than background radiation (Righi et al. 2000). Earlier studies have reported significant activity concentrations of radionuclides such as ^238^U, ^232^Th, and ^226^Ra in fertilizers and the area around fertilizer plants (Bolivar et al. 2000). Natural radionuclides are present in various geological formations, such as soil, water, sediments, and building materials widely distributed in the environment. When rocks undergo natural processes, radionuclides are transported to the soil by rainfall and run-off (Taskin et al. 2009). However, some rocks are found at higher concentrations than others; for example, felsic igneous rocks are found at higher concentrations than sedimentary rocks (Bastos and Appoloni 2009).

Radioactive contamination, occurring when radionuclides are discharged into an aquatic system and eventually settle to the bottom by sorption on suspended solids, stands out as one of the most hazardous forms of pollution (Eisenbud and Gesell 1997). However, it is necessary to understand the water body’s morphology, the ecological significance of aquatic systems concerning environmental variables, particularly the distribution of species and variety and the influential relationship between the various parts of the food chain. Diatoms, an important category of primary producers in aquatic ecosystems and an essential component of the phytobenthos, are excellent indicators of eutrophication. They are also radionuclide indicators in aquatic ecosystems (Pradhan and Sukla 2019; Banerjee et al. 2022).

The Ismailia Canal, which crosses between residential areas and some factories, is considered one of the crucial branches of the Nile in Egypt. However, several sources of pollution influence the Ismailia Canal’s water quality (Geriesh et al. 2008), owing to several effluents that can significantly alter the chemical and physical characteristics of the River Nile and its canals. This, in turn, affects the ecological status of river breaches (El-Sayed 2011) and may change the radionuclides concentration in this area (Abdel Malik et al. 2010). Due to these circumstances, continuous monitoring of the ecological characteristics and radioactivity levels of the water in the Ismailia Canal is essential to ensure its safety. Therefore, the current study aimed to assess the water quality, the radioactive contamination in water and sediments, and the biodiversity of primary producers presented by the attached diatoms. This evaluation seeks to evaluate the potential of diatoms for bio-indication in the Ismailia Canal. Furthermore, the study aims to calculate the environmental impact and hazard indices of industrial pollutants in the water body of the canal. This monitoring is crucial to determining the variation of environmental characteristics and radioactivity levels resulting from the industrial effluents in the Ismailia Canal.

## Materials and methods

### Study area

**Fig. 1:**
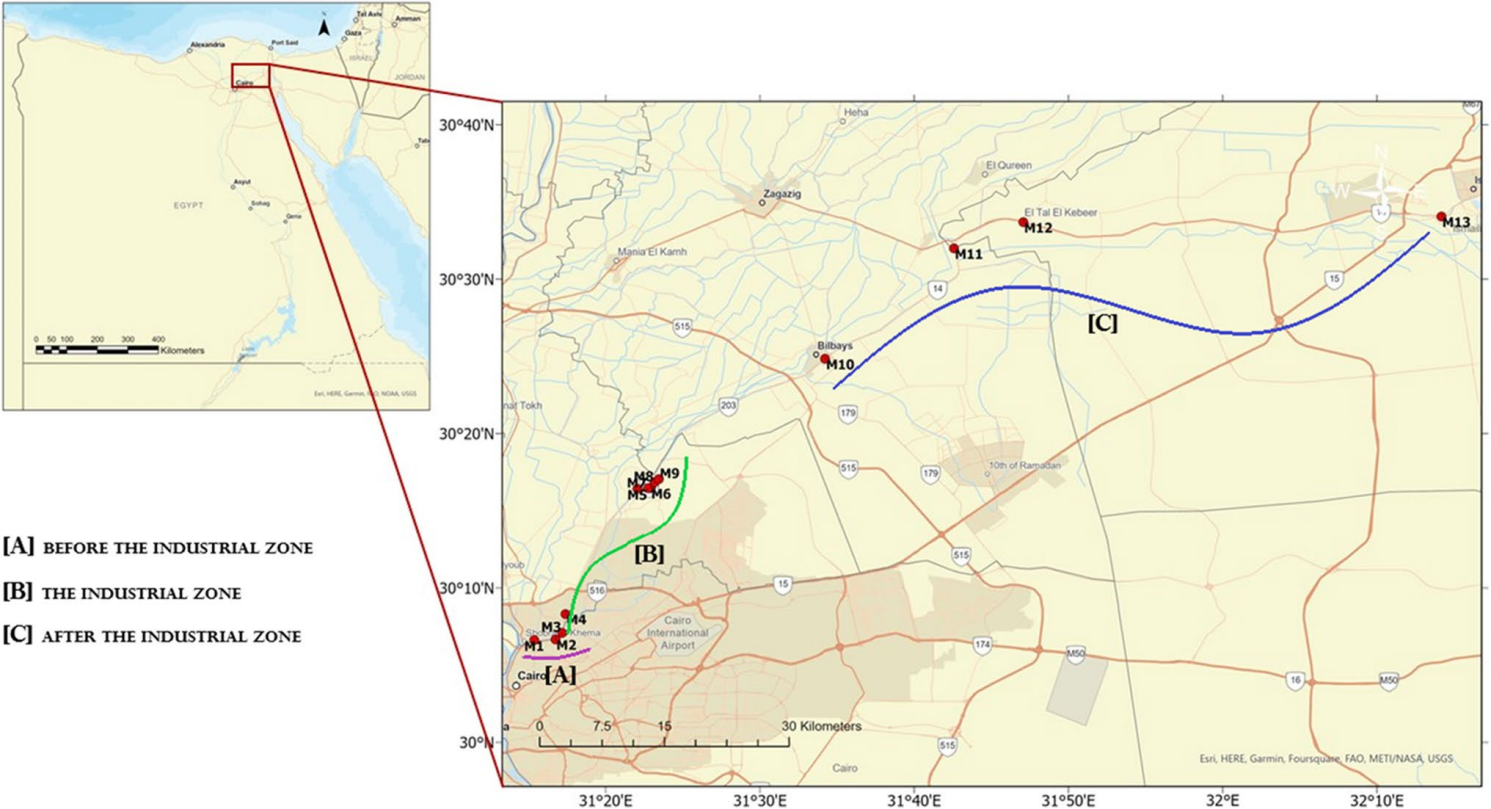
Location map of the sample sites at Ismailia Canal

The Ismailia Canal is one of Egypt’s crucial irrigation and water resources. It was built in 1862 to supply drinking water to the cities in the Suez Canal area. Stretching approximately 125 km, it is located east of the Nile at Shupra, north of Cairo. Characterized by a depth ranging from 1 to 3 m and a width ranging from almost 30 to 70 m, with a mean water flow of 0.28 m/s (Geriesh et al. 2008) (Fig. 1). The canal passes through different geologic formations, including silt and mud on its upstream west bank and sand and marl on its downstream east bank (El-Mathana et al. 2021). Around 12 million Egyptians rely on it as their primary source of drinking water and irrigation (Geriesh et al. 2008). The outflow of the canal is about 5,000,000 m^3^ of water daily for drinking, irrigation, and industrial purposes (El-Haddad 2005). Major industrial zones line the upstream section from Cairo to Abu Za’baal (Shupra El Kheima, Musurod, and Abu Za’baal industrial zones). Notably, at the field scale, Al Delta Company for Iron, the Electro Cable Egypt Company (ECE), the Petroleum Company and the Aluminium Sulfate Company serve as point sources of pollution. The Abu Za’baal Fertilizers Company acts as a point and non-point source of pollution. In the area from Abu Za’baal to El-Ismailia along the canal’s banks, many agricultural run-offs are classified as non-point sources of contamination. Toxic metals and wastes from factories and residential areas enter the canal. Farmers employing many fertilizers also risk leakage into the River Nile, its canal, and its branches (Manigandan and Chandar Shekar 2014). Superphosphate and rock phosphate stand as the primary sources of phosphate fertilizers used in agricultural production in Egypt, with the recent adoption of alternatives like phosphoric acid for production (Mohamed 2021). According to Atlas, the average annual rate of fertilizer use in Egypt has increased by 3% per hectare from the 1970s to the 2020s. An elevated level of some toxic metals, such as cadmium and lead, surpassing permissible limits in the surface water of the Ismailia Canal, may be attributed to the waste of electric batteries, electronic components, and industrial wastes (Ibrahim et al. 2014). Indeed, the industrial discharge pipes of the Abou-Zabal (AZ) Company, sulfate of aluminium and potassium (alum, p), iron and steel companies, and ceramic factories constitute point sources of contamination.

### Sampling collection and preparation

The sediment and water sampling strategy involves selecting points at the pollution discharge site, downstream and upstream of the discharge. The sediment and water samples were collected from 13 locations, as described in Table 1, along the length of the Ismailia Canal. Three samples from each of these sites were carefully selected and intended to be representative of each site. Environmental parameters, including pH value, electrical conductivity, and water temperature, were measured in situ using the Hydrolab model (Multi Set 430i WTW). A Secchi disk (30 cm in diameter) was used to measure transparency.

**Table 1:** Details, latitude, and longitude of sampling locations

Sample code		Site location	Site descriptions	Latitude	Longitude
Before the industrial zone [A]	M1	El Mazalate (Mouth of Ismailia Canal)	Around the civilization area in the vicinity of Cairo	30° 06ʹ 628″	31° 15ʹ 365″
	M2	Close to Al-Amiria Water Treatment Plant	Received the discharge of waste water from the water treatment plants	30° 06ʹ 672″	31° 16ʹ 744″
The industrial zone [B]	M3	Mostourd (Izbat El Rayse) (in front of Al Delta company for Iron, Electro Cable Egypt Company (ECE))	Received the industrial waste from the company of the power cables and telecommunication cables as well as metal wires	30° 07ʹ 097″	31° 17ʹ 165″
	M4	Downstream of Petroleum Company (Drain)	Received the industrial waste from direct discharge of petroleum and natural gas activities	30° 08ʹ 315″	31° 17ʹ 398″
	M5	Upstream of Abu Za’baal fertilizer Company	Received atmospheric deposition from dust from the Abu Za’baal Fertiliser Company, industrial waste of the company and agricultural run-off	30° 16ʹ 094″	31° 22ʹ 043″
	M6	In front of Abu Za’baal fertilizer Company	Received atmospheric deposition from dust from the Abu Za’baal Fertiliser Company, industrial waste of the company and agricultural run-off	30° 16ʹ 466″	31° 22ʹ 887″
	M7	Downstream of Za’baal fertilizer Company (Drain)	Received atmospheric deposition from dust from the Abu Za’baal fertilizer company, industrial waste of the company and agricultural run-off	30° 16ʹ 471″	31° 22 741″
	M8	Close to Aluminum Sulfate Company (Drain)	Received the chemical waste from the Aluminum Sulfate Company and agricultural run-off	30° 16ʹ 843″	31° 23ʹ 165″
	M9	Downstream of Aluminum Sulfate Company	Received the chemical waste from the Aluminum Sulfate Company and agricultural run-off	30° 17ʹ 050″	31° 23 443″
After the industrial zone [C]	M10	Bilbeis	Agricultural run-off	30° 24ʹ 840″	31° 34ʹ 225″
	M11	Al Abbas	Agricultural run-off	30° 31ʹ 984″	31° 42ʹ 576″
	M12	El-Tal El-Kabee	Agricultural run-off	30° 33ʹ 690″	31° 47ʹ 068″
	M13	El-Ismailia (before bifurcation)	Agricultural run-off	30° 34ʹ 057″	32° 14ʹ 178″

Regarding the radioactivity measurements, bottles were washed with diluted HCl acid to prevent radioactive materials from precipitating on the surface. The samples were collected using a water sampler in a 5-l polyvinyl chloride plastic container without filtration and stored in an icebox until we returned to the lab. Surface sediment is sampled using an Ekman grab sampler (15 cm × 15 cm, 225 cm^2^), quickly packed in airtight polythene bags, and stored in an ice box at the site. The sediment samples would be crushed, dried at 70 °C, and sieved to a mesh size of 63 mesh. Commercial fertilizer samples were collected from the local market, and raw materials were obtained from the Abou-Zabal (AZ) Company. Sampling occurred during the winter and summer of 2022 (M1-M13) (Fig. 1, Table 1). For diatom examination, epipelic samples were taken from the sediment’s surface layer (nearly 1 cm or within the first millimetres), where most benthic diatoms are present. This community was quantitatively collected by inserting a spatula into glass vials under a defined sediment surface area to a depth of 2 cm.

### Chemical analysis

Water samples were stored in 26 polyethylene bottles in an ice box for laboratory analysis. The American Public Health Association (APHA, American Public Health Association 1995) explained how to determine most physicochemical parameters. The total dissolved solids (TDS) were determined by filtrating a sample volume with a glass microfiber filter (GF/C), and a known filtrate volume was evaporated at 105 °C. The total suspended solids (TSS) were measured by the filtration of a specific sample volume and subtraction of the TS-TDS. Total solids (TS) were determined by the evaporation of a certain amount of sample that had been well mixed. The modified Winkler method measured dissolved oxygen (DO), while chemical oxygen demand (COD) was estimated according to the potassium permanganate method. A 5-day method was used to determine biochemical oxygen demand (BOD). Transparency was measured using a white/black Secchi disk (25 cm in diameter) and expressed as the Secchi disk depth (SDD). Various parameters, including pH, temperature (°C) of water, and conductivity (EC, µS/cm), were in situ measured using a hydrolab model (Multi Set 430i WTW, Weilheim, Germany) after previous calibration. The chloride content was determined using the Mohr method, and the sulfate content was determined by the turbidimetric method. Indicators such as methyl orange and phenolphthalein were used to measure water alkalinity immediately after sampling. Magnesium and calcium were measured by direct titration in an EDTA solution, and K^+^ and Na^+^ were measured using a ‘Jenway PFP7 UK flame photometer. A blank and calibration standard curve was prepared in a stepped amount in the following applicable ranges: 1, 2, 5, 100, and 200 mg/l, with concentrations deduced from the calibration curve from a series of standard solutions. A Jenway 6800 double-beam spectrophotometer, UK (scan in the range 190 to 1100 nm with resolutions up to 0.1 nm and scan speeds up to 3600 nm/s) and colorimetric methods were used to measure NO_2_-N, NO_3_- N, NH_4_- N, PO_4_-P, and SiO_4_ concentrations. Total phosphorus (TP) was determined as reactive phosphate after persulfate digestion. All solutions and chemicals used in this study were of the highest purity (Sigma-Aldrich Analytical Grade).

### Radioactivity measurements

Non-destructive sodium iodide gamma spectrometry using a 2 × 2 NaI(Tl) setup and a multichannel analyser (MCA) was used to measure radioactivity in the samples (water and sediment): The resolution (FWHM) is 24.22 keV at 1.33 MeV ^60^Co, and the relative efficiency is 7% with a lead shield to reduce the background. The sample was on the detector for at least 72 h, and the spectra were analysed manually with a spreadsheet (Microsoft Excel) for radionuclide determination and automatically with the Maestro computer program (EC&G ORTEC). The daughters (^214^Pb and ^214^Bi) were used to determine the activity concentration of the ^238^U(^226^Ra) series over 295.1 keV and 352.0 keV for ^214^Pb and 609.3 keV, 1120.3 keV, and 1764.5 keV for ^214^Bi. The activity concentration of the ^232^Th series was calculated using the gamma energy lines 911.2 keV and 969.11 keV for the ^228^Ac and 2614.4 keV for the ^208^Tl and 239 keV for the ^212^Pb and using the direct gamma energy line 1460.8 keV for the measured ^40^K (Chieco et al. 1990). The calibration of the detector efficiency was performed via the IAEA standard sources RGU-1, RGTh-1, and RGK-1 (IAEA 1987). The IAEA-certified reference materials (IAEA-443, IAEA-446, IAEA-410, and IAEA-312) are used to perform quality control (QC) procedures to verify the reliability and precision of the experimental results reported in this study (Appendix Table 10). After that, the samples were stored for at least 4 weeks in sealed beakers. This ensured that there was no radon loss and that radium isotopes and their daughters had reached a secular equilibrium. The activity concentrations of the radionuclides, expressed as becquerels per kilogram for sediment and becquerels per litre for water samples, were determined using the formula developed by El Afifi et al. (2006). The precision of the gamma spectrometer is determined by the lowest detection limits (LLDs) of the measuring system (USDOE 1992). The LLD values obtained for ^40^K, ^226^Ra, and ^232^Th were 8, 1, and 1 Bq/kg, respectively, for the sediment samples and 0.5, 0.04, and 0.05 Bq/l, respectively, for the water samples. The uncertainty of activity, U_AC_, is calculated from uncertainty of components C, P, and W as follows (Asaduzzaman et al. 2016):

1 $${{\text{U}}}_{{\text{AC}}}=\sqrt{{\lceil\frac{{U}_{{\text{C}}}}{{{\text{N}}}_{{\text{C}}}}\rceil}^{2}+{\lceil\frac{ {U}_{\upvarepsilon }}{\upvarepsilon }\rceil}^{2}+{\lceil\frac{{U}_{{\text{p}}}}{{\text{P}}}\rceil}^{2}+{\lceil\frac{{U}_{w}}{{\text{W}}}\rceil}^{2}}$$

where ${U}_{{\text{C}}}$,${U}_{\upvarepsilon }$, ${U}_{{\text{P}}}$, and *U*_W_ are respective uncertainties for the net count per second, the detector efficiency, the gamma decay transition probability, and the sample mass.

### Diatom sample preparation and laboratory analysis

After adding tap water, the preserved samples were placed in a 500-ml beaker and vigorously stirred for a few minutes. Heavy clay and sand particles were allowed to settle in the samples briefly following stirring. At the same time, the supernatant fluid containing microalgae was put into a cylinder with a capacity of 1 l. This process was repeated at least six to eight times. A few drops of an acidic solution of iodine were also added. The samples were concentrated to make 100 ml of samples in plastic bottles. Homogenized samples, consisting of 0.5 g of clay sediment and approximately 2 g of sandy sediment, were subjected to concentrated nitric and sulfuric acid treatments. The samples were heated until all organic matter was oxidized, according to ANS (2002), for further identification confirmation. Samples were neutralized by repeated washing and concentration (Belal 2012). Utermohl (1958) approach was employed for diatom counting and identification using an inverted microscope (Zeiss, Model Axiovert 25C). Primary sources for identifying epiphytic diatoms included Cleve-Euler (1953), Patrick and Reimer (1975), and Krammer and Lange-Bertalot (1986, 1988, and 1991).

### Environmental impact and hazard indices

#### Water quality index (WQI)

The water quality of the Ismailia Canal was assessed using the CCME-WQI module and calculated using the following equation (CCME 2001):

2 $${\text{CWQI}}=100-\frac{\sqrt{{F}_{1}^{2}+{F}_{2}^{2}+{F}_{3}^{2}}}{1.732}$$

*F*_1_ (scope) is the percentage of variables that do not meet their objectives at least once during the considered period (failed variables). *F*_2_ (frequency) is the percentage of individual tests that do not satisfy their objectives. *F*_3_ (amplitude) represents the amount of failed test values that do not meet their targets. Water quality is classified according to one of the categories outlined in Appendix Table 11. The guidelines from the Egyptian Drinking Water Quality Standards (2007) were used to calculate the WQI for drinking water. The WQI for irrigation water was calculated following the FAO (1994) guidelines. Additionally, the guidelines of CCME 2007 were used to calculate the protection of aquatic life. Detailed water quality guidelines are illustrated in Table 2.

**Table 2:** Mean, standard deviation and range of water parameters compared to guidelines used in WQI

Parameters	Range	Mean ± SD	CV %	Drinking water		Irrigation^3^	Aquatic life^4^
				Egypt^1^	WHO^2^		
Temp, °C	15.30–37.22	24.41 ± 7.19	29.48			< 35	8.0–28.0
Trans, cm	40–140	86.67 ± 31.44	36.27				
EC, mS/cm	293–530	373.08 ± 74.50	19.97	2000		3000	
pH	7.31–8.2	7.83 ± 0.22	2.85	6.5–8.5	8.5	8.5	6.5–9
TDS, mg/l	175–317.8	225.79 ± 43.15	19.11	1000	500	2000	< 500
TSS, mg/l	9.22–69	33.12 ± 14.17	42.79				25
TS, mg/l	307.30–534	391.38 ± 56.40	14.41				
DO, mg/l	2.8–7.10	5.38 ± 1.03	19.13	6		> 4	> 5.5
BOD, mg/l	0.40–5.80	2.62 ± 1.69	63.33	3			< 6
COD, mg/l	2.00–12.40	5.15 ± 2.89	55.98	10	10		7
NH_4_, µg/l	107.64–157.68	134.98 ± 14.10	10.45	450	200	5000	1370
NO_2_, µg/l	0.012–9.91	3.01 ± 2.29	76.02	5	900		60
NO_3_, µg/l	81.37–198.36	142.01 ± 31.56	22.23	10,000	11,000	10,000	2930
PO_4_, µg/l	24.23–407.85	77.41 ± 100.10	129.32			2000	
TP, µg/l	137.22–524.25	237.01 ± 96.61	40.76	1000			
SiO_4_, mg/l	0.58–5.43	2.34 ± 1.47	60.65				
CO_3_, mg/l	0.00–10.47	7.20 ± 3.38	47.00			3	
HCO_3_, mg/l	143–185.11	159.49 ± 10.86	6.81			610	
Cl, mg/l	26.99–31.99	28.15 ± 3.44	12.21	250	200	1063	120
SO_4_, mg/l	26.55–79.19	36.71 ± 13.16	35.83	250	250	960	
Ca, mg/l	16.03–21.64	18.62 ± 1.31	7.03	75	75	400	
Mg, mg/l	4.86–9.73	7.24 ± 1.38	19.03	50	50	60	
Na, µg/l	22.45–34.66	27.81 ± 3.44	12.35	200		919	
K, µg/l	6.5–9.36	7.92 ± 0.85	10.72			2	

^1^Egypt (2007)

^2^WHO (2011)

^3^FAO (1994)

^4^CCME (2007)

#### Radiation hazard indices

Safely assessing the health risks of exposure to humans or the environment requires using many standard radiation hazard indices. The estimated indices include the absorbed dose rate in the air (D) (UNSCEAR 2000), annual effective dose rate (AED) (UNSCEAR 2000), radium equivalent activity (Ra_eq_) (Kurnaz et al. 2007), the external hazard index (*H*_ex_) (UNSCEAR 2000), and the representative level index (*Iγ*) (Chad-Umoren and Umoh 2017). The calculations for these indices are detailed in Appendix Table 12. To estimate the total risk assessment of consumed water, it is crucial to consider the health impacts of the hazards connected to all radium isotopes, such as the cancer risk (EPA 1999; Abdellah and Diab 2012) and the effective radiation (DRW) (USEPA 1999), using the equations provided in Appendix Table 12. These assessments are essential for determining any potential health risks.

### Statistical and data analysis

The correlation between diatom diversity, richness, evenness, and physicochemical and radionuclide properties was assessed by calculating the Pearson correlation coefficient using Primer software version 5 (Clarke and Gorley 2001). The Pearson correlation coefficient (*r*) quantifies the strength of a linear association between diatoms (richness, evenness, and diversity) and the physicochemical and radioactive elements. This analysis aimed to determine whether these measured variables influenced the components of the diatom communities in the Ismailia Canal. A redundancy analysis (RDA) was also performed as a constrained ordination to ascertain how variance in the diatom community composition could be explained by the variation in the measured chemical, physical, and radioactive elements. Using Canoco Windows version 4.1 (Ter Braak 1987), the relative abundance of frequently recorded diatom taxa (≥ 30%) was incorporated alongside environmental variables. The Canoco 4.5 software (Ter Braak and Smilauer 2002) was used to perform the discriminant analysis (DA) and the redundancy analysis (RDA) of the physicochemical variables, nutrient salts, and significant elements for the different locations, including the radioactive elements in the Ismailia Canal. A Monte Carlo permutation test with 499 permutations was set to identify the significant environmental variables, major elements, and nutrient salts influencing the distribution of radioactive elements (*P* < 0.05).

## Results and discussion

### Chemical analysis

Table 2 illustrates water quality parameters. The data shows low regional variations in water temperature, except in thermally polluted regions such as station (3), which recorded the highest summer temperature of 37.22 °C. Transparency was affected by the industrial effluent discharge, particularly at stations 2, 4, 6, and 8 varying between 40 and 140 cm, with a highly significant geographical and temporal variations (*P* < 0.01). In the present study, transparency exhibited a negative correlation with TSS and TDS, which agrees with those reported by Goher et al. (2014), who reported similar negative associations between cations and anions, TSS and TDS, and transparency in Ismailia Canal water. It was noticed that an increase in the canal’s flow rate led to an increase in the water level, causing sediment agitation, an elevation in the concentration of TSS, and, consequently, a decrease in the water’s transparency. A high temporal difference (*P* < 0.01) was noted for TSS and TDS.

The pH ranged from 7.31 to 8.2, remaining within the limits for drinking water and aquatic life guidelines in two seasons (Abdel-Aal et al. 2023). Elevated electrical conductivity (EC) values were observed at the stations (2, 4, 6, and 8) nearest the industrial discharges, while lower values were observed at the stations upstream and downstream of the pollution sources. In the winter, the lower water level of the Ismailia Canal led to increased ions concentration, raising the EC level, which agrees with the results obtained by Hussein et al. (2023), Talab et al. (2016), and Goher et al. (2015) in the Nile River, the El-Rayah of Nile, and El-Bahr El-Pharaony, respectively. TDS levels are negatively correlated with water flows, with winter recording the highest levels and summer recording the lowest levels (high dilution). El Sayed et al. (2023) and Goher et al. (2014) reported that bicarbonate is the leading cause of the Ismailia Canal’s total alkalinity. Highly significant variations (*P* < 0.01) in bicarbonate alkalinity were observed between the sampling seasons. At the same time, spatial variation (*P* < 0.05) was recorded along the sampling sites of the canal, except for the polluting point sources (2, 4, 6, and 8). A negative significant correlation (*r* =  − 0.66, *n* = 26) (*P* < 0.05) was observed between HCO_3_^−^ and silicate concentrations, potentially attributed to algal uptake of CO_2_, with silicate and CO_2_ being absorbed by diatoms (Wang et al. 2013). Dissolved oxygen (DO) is critical for assessing the water conditions for aquatic life and drinking water (El Sayed et al. 2020). Mean DO values remained within the limits recommended by CCME (2007) for aquatic life at all sites except station (10) (in front of the source of domestic waste). The BOD and COD values showed significant spatial variations (*P* < 0.01), ranging from 0.40 to 5.80 mg/l and 2.00 to 12.40 mg/l, respectively. BOD and COD are generally indicators of organic pollution (Elsayed et al. 2019; Goher et al. 2021). The discharge of industrial effluents was responsible for the maximum COD value measured at the station (8) during two seasons, consistent with the results obtained by Hussien et al. (2023) in the Nile River, El Sayed et al. (2023) in the Ismailia Canal, and Talab et al. (2016) in the Nile Rayahs.

The distribution of chloride and sulfate exhibited significant temporal differences (*P* < 0.01), with significant increases at the site (6), consistent with the findings of Abdo and El-Nasharty (2010). Sulfate and chloride have a positive correlation with Ca^+2^ and Mg^+2^. Calcium and magnesium values varied from 16.3 to 21.64 and 4.86 to 9.73 mg/l, respectively, with a substantial seasonal variation (*P* < 0.01).

Calcium and magnesium concentrations were at their lowest during the summer season. This could be attributed to the adsorption of clay minerals and deposition on the bottom due to temperature increases, as El Bouraie et al. (2010) suggested, and the consequences of the flood period. Sodium and potassium levels were recorded at 22.45–34.66 and 6.5–9.36 mg/l, respectively, with significant variations between sites and seasons (*P* < 0.01). Nutrient salts were within the ranges of 0.012–9.91, 81.37–198.36, 107.64–157.68, 24.23–407.85, 137.22–524.25 µg/l, and 0.58–5.43 mg/l for nitrite, nitrate, ammonia, orthophosphate, total phosphorus, and silicate, respectively. The highest concentrations of nutrient salts were found at the site (6), likely attributed to the industrial effluents discharged.

### Radioactivity measurement

#### Water

**Fig. 2:**
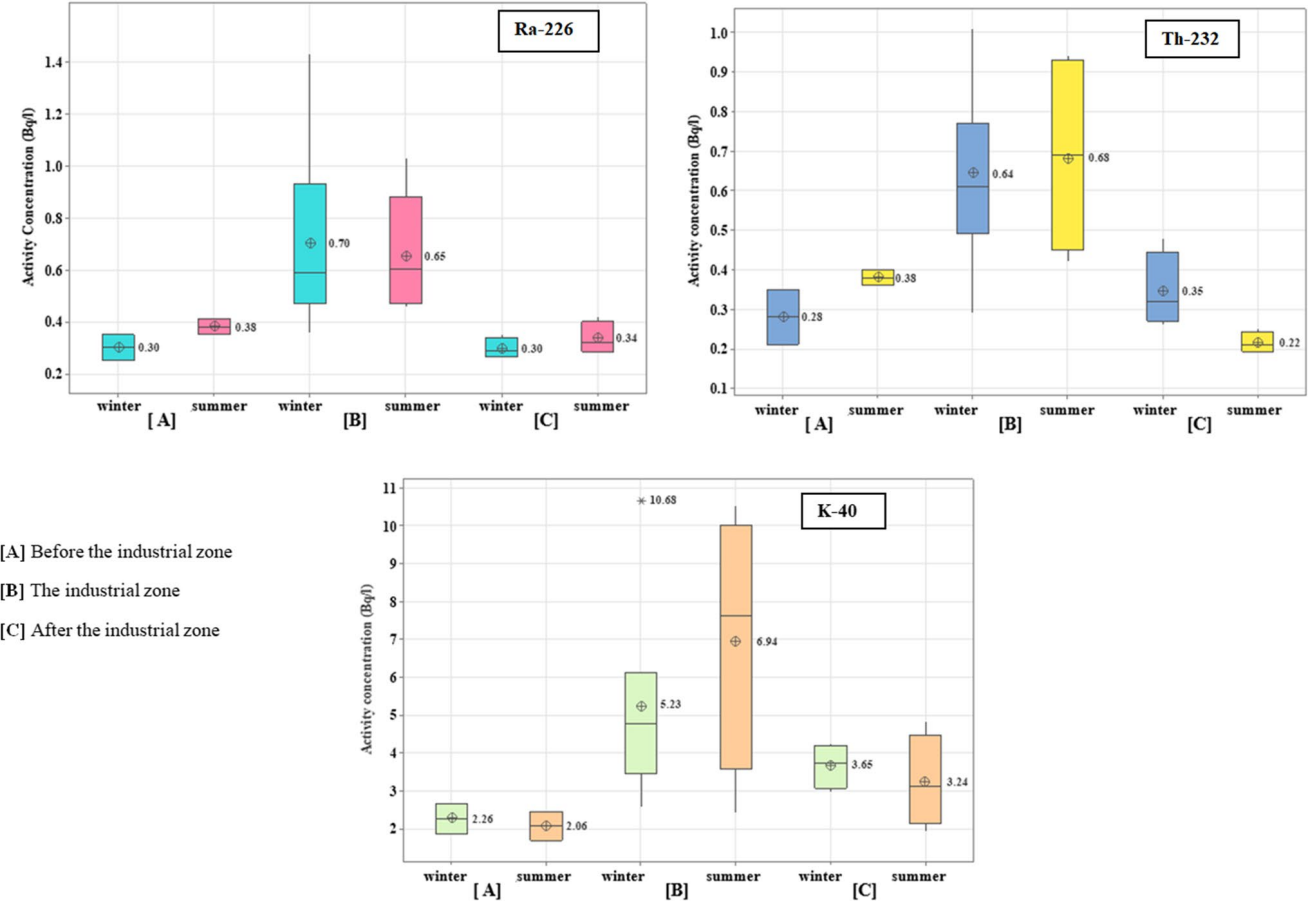
The distribution of ^226^Ra-series, ^32^Th-series, and ^40^K in water samples of Ismailia Canal

The activity concentration of Ra-226 series, Th-232 series, and K-40 in water samples during the winter and summer seasons is presented in Fig. 2 and Table 3. In winter, the specific activity of ^226^Ra, ^232^Th, and ^40^K varied from 0.25 ± 0.05 to 1.43 ± 0.22 Bq/l, 0.21 ± 0.09 to 1.01 ± 0.16 Bq/l, and 1.85 ± 0.25 to 10.68 ± 1.39 Bq/l, respectively. In summer, the range was 0.28 ± 0.04 to 1.03 ± 0.14 Bq/l, 0.19 ± 0.02 to 0.94 ± 0.05 Bq/l, and 1.67 ± 0.18 to 10.53 ± 1.21 Bq/l, respectively. The canal is divided into three zones: before the industrial zone [A], the industrial zone [B] (including the Petroleum Company, the Delta Company for Iron, Aluminium Sulfate Company, the Abou-Zabal (AZ) Company, and sulfate of aluminium and potassium company), and after the industrial zone [C]. The ^226^Ra, ^232^Th, and ^40^K distributions over two seasons were almost identical in zones [A] and [C], which is compatible with the distribution pattern of the elements in the River Nile (El-Gamal et al. 2019a, b). However, the mean specific activity of ^226^Ra, ^232^Th, and ^40^K in zone [B] (the industrial zone) was more than twice that of zones [A] and [C]. Significant variations in the activity concentrations of ^226^Ra, ^232^Th, and ^40^K were observed in the industrial region, with an increase in the activity concentration of ^232^Th and ^40^K during the summer and a rise in ^226^Ra during the winter. The mean activity concentration of ^226^Ra decreased with increasing sampling distance from the industrial zone [B], which includes the Abu Za’baal Fertiliser Factory, the Petroleum Company, the Delta Company for iron, and the Aluminium Sulfate Company.

**Table 3:** The activity concentration of radionuclides in water samples (Bq/l), sediment samples, commercial fertilizers, and raw materials (Bq/kg) (activity ± total error)

Sample code	Water							Sediment					
	Ra-226 series			Th-232 series		K-40		Ra-226 series		Th-232 series		K-40	
	Winter		Summer	Winter	Summer	Winter	Summer	Winter	Summer	Winter	Summer	Winter	Summer
Before the industrial zone [A]	M1	0.25 ± 0.05	0.35 ± 0.03	0.35 ± 0.11	0.4 ± 0.09	2.66 ± 0.26	2.44 ± 0.18	20.78 ± 0.98	10.18 ± 0.59	28.09 ± 1.63	5.80 ± 0.50	190.56 ± 4.03	150.45 ± 3.27
	M2	0.35 ± 0.09	0.41 ± 0.08	0.21 ± 0.09	0.36 ± 0.08	1.85 ± 0.25	1.67 ± 0.18	25.51 ± 1.60	15.52 ± 0.69	10.87 ± 1.15	18.86 ± 1.62	244.31 ± 6.29	320.45 ± 8.06
The industrial zone [B]	M3	0.60 ± 0.03	0.47 ± 0.09	0.49 ± 0.06	0.42 ± 0.07	3.53 ± 0.05	5.78 ± 0.73	25.12 ± 1.73	29.56 ± 1.41	14.05 ± 1.31	18.28 ± 1.14	296.22 ± 8.54	251.09 ± 5.48
	M4	0.59 ± 0.09	0.46 ± 0.04	0.60 ± 0.14	0.69 ± 0.10	4.77 ± 0.11	2.40 ± 0.19	23.09 ± 1.19	24.93 ± 1.33	14.62 ± 1.02	13.67 ± 1.33	309.12 ± 5.75	182.68 ± 6.22
	M5	0.54 ± 0.10	0.60 ± 0.04	0.29 ± 0.08	0.45 ± 0.06	2.56 ± 0.42	3.56 ± 0.34	28.72 ± 1.26	27.4 ± 1.54	23.20 ± 2.99	12.58 ± 1.16	424.36 ± 16.44	270.20 ± 6.38
	M6	0.93 ± 0.06	1.03 ± 0.14	1.01 ± 0.16	0.94 ± 0.05	6.10 ± 0.56	10.53 ± 1.21	35.13 ± 2.01	30.01 ± 1.67	17.66 ± 1.28	10.55 ± 1.33	332.33 ± 7.48	272.7 ± 6.95
	M7	1.43 ± 0.22	0.88 ± 0.08	0.74 ± 0.13	0.93 ± 0.12	10.68 ± 1.39	10.00 ± 0.83	41.16 ± 0.79	35.45 ± 0.61	14.75 ± 0.99	9.26 ± 0.47	333.32 ± 4.66	257.22 ± 2.72
	M8	0.36 ± 0.07	0.52 ± 0.07	0.61 ± 0.09	0.76 ± 0.11	5.52 ± 0.68	8.69 ± 0.77	61.07 ± 3.56	39.48 ± 1.50	29.63 ± 2.5	17.58 ± 1.16	590.01 ± 13.21	375.96 ± 5.02
	M9	0.47 ± 0.03	0.60 ± 0.05	0.77 ± 0.1	0.59 ± 0.07	3.45 ± 0.3	7.61 ± 0.54	54.15 ± 1.19	41.1 ± 1.17	7.8 ± 0.68	13.28 ± 1.09	363.45 ± 8.80	351.13 ± 7.59
After the industrial zone [C]	M10	0.35 ± 0.04	0.42 ± 0.04	0.48 ± 0.12	0.20 ± 0.04	4.23 ± 0.68	1.91 ± 0.10	25.62 ± 1.14	23.66 ± 0.70	8.75 ± 0.7	11.37 ± 1.05	165.21 ± 3.35	116.97 ± 2.62
	M11	0.3 ± 0.04	0.34 ± 0.05	0.34 ± 0.12	0.19 ± 0.02	4.03 ± 0.65	4.80 ± 0.61	26.82 ± 1.62	19.60 ± 0.94	14.92 ± 1.19	10.78 ± 0.78	281.91 ± 7.42	139.35 ± 3.80
	M12	0.28 ± 0.02	0.3 ± 0.04	0.3 ± 0.12	0.22 ± 0.02	3.39 ± 0.39	2.78 ± 0.41	22.55 ± 2.17	25.62 ± 0.75	11.48 ± 1.16	11.32 ± 0.52	141.04 ± 7.36	203.71 ± 3.26
	M13	0.26 ± 0.02	0.28 ± 0.04	0.26 ± 0.02	0.25 ± 0.06	2.94 ± 0.1	3.45 ± 0.50	14.19 ± 1.21	17.48 ± 0.41	14.66 ± 1.20	13.36 ± 0.78	134.40 ± 5.15	98.48 ± 2.75
Mean (Canal)	0.52 ± 0.07		0.51 ± 0.06	0.50 ± 0.1	0.49 ± 0.07	4.29 ± 0.45	5.05 ± 0.51	31.07 ± 1.57	26.15 ± 1.02	16.19 ± 1.37	12.82 ± 0.99	292.79 ± 7.58	230.02 ± 4.93
Worldwide (WHO 2003; IAEA 1996)	1			1		4		35		35		370	
Phosphate fertilizer samples													
Sample code				Ra-226 series		Th-232 series		K-40					
Granulated single super phosphate (SSP)	Commercial fertilizers			268.11 ± 2.62		19.48 ± 1.27		241.24 ± 4.28					
Phosphorus calcium sulfate (PCS)				99.64 ± 2.06		22.51 ± 1.52		406.66 ± 5.49					
R1	Raw materials			362.61 ± 2.53		25.19 ± 1.12		327.04 ± 4.62					
R2				390.23 ± 2.91		23.56 ± 1.7		310.21 ± 4.02					
Soil sample	Collected 100–150 m from fertilizer company			73.2 ± 2.5		18.51 ± 0.98		350.23 ± 4.98					
Control sediment sample (River Nile sample)				26 ± 1.6		19 ± 1.5		205 ± 4.5					

Radium is less common than other alkaline earth metals due to its low natural abundance. It exists as Ra ^2+^, which is poorly soluble in natural water (Chu and Wang 2000), often precipitating in sulfate, carbonate, and chromate salts (Edsfeldt 2001). Additionally, Ra-226 decays by alpha particle radiation into radon-222, a gas with a high probability of decaying in the body when inhaled, emitting alpha particle radiation in the body. The long-term exposure of people to high concentrations of radon and its progeny in mines and phosphate factories causes pathological effects, including functional changes in the respiratory system and lung cancer (Dajawa et al. 2009). Low values of ^226^Ra near the Aluminium Sulfate Company at stations M8 and M9 could result from radium precipitation from water due to seepage of sulfate from the Aluminium Sulfate Company. In the same context, the ^232^Th and ^40^K have a significant impact with higher activity concentrations in the industrial zone compared to the rest of the canal, especially during the summer. Thorium is particularly insoluble in natural waters and tends to be combined with solid materials (El-Reefy et al. 2010).

Furthermore, thorium is challenging to transfer in any environment due to the strong stability of the insoluble oxide ThO_2_ (Mamoney and Khater 2004). Modest seasonal variation in ^40^K concentrations, rising during the summer (season of agriculture), may be due to dissolved potassium due to the increased water flow. The natural radioactivity of most of the sampling stations of the canal during two seasons falls within the permissible limits of natural radionuclides, except for some stations, such as M6 and M7, exhibiting higher natural radioactivity than the worldwide value of drinking water (IAEA 1996; WHO 2003). This may be due to the accumulation of phosphate dust and phosphorus ore and processing waste discharging into the canal near the canal’s shoreline.

#### Sediment

**Fig. 3:**
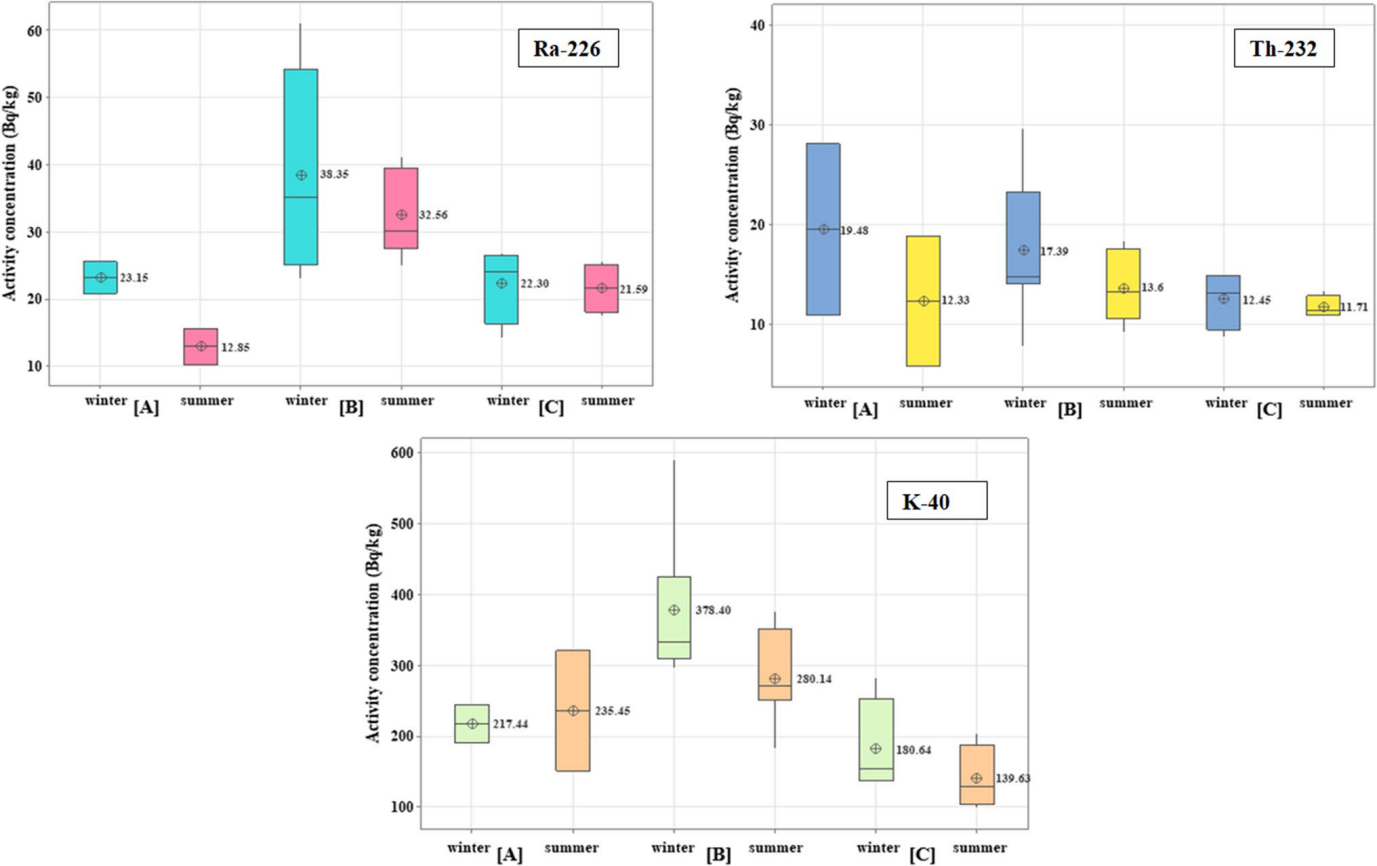
The distribution of ^226^Ra-series, ^232^Th-series, and ^40^K in sediment samples of Ismailia Canal

The specific activity concentration of ^226^Ra, ^232^Th, and ^40^K in sediment was determined in becquerel per kilogram during two seasons, as illustrated in Fig. 2 and Table 3. The activity of radionuclides for ^226^Ra, ^232^Th, and ^40^K varied from 14.19 ± 1.21 Bq/kg at M13 to 60.01 ± 3.56 Bq/kg at M8, 7.8 ± 0.68 Bq/kg at M9 to 29.63 ± 2.5 Bq/kg at M8, and 134.4 ± 5.15 Bq/kg at M13 to 590.01 ± 13.21 Bq/kg at M8, respectively, during the winter. In summer, the range was 10.18 ± 0.59 to 41.1 ± 1.17 Bq/kg, 5.80 ± 0.50 to 18.86 ± 1.62 Bq/kg, and 98.48 ± 2.75 to 375.96 ± 5.02 Bq/kg, respectively. Our findings indicated that the ^226^Ra concentration in zones [A] and [C] falls within the typical range of unpolluted surface sediments (20–30 Bq/kg) for ^226^Ra concentration (Bolivar et al. 1996), but the activity concentration of ^226^Ra was higher three times in the sediments of zone [B]. This enhancement was concentrated noticeably close to the canal discharge locations for the Abu Za’baal Fertilizer & Chemical Company and Aluminium Sulfate Company. The wastes from processing phosphate and phosphate raw materials uploaded into the canal side from loaders and transported over the road by conveyor belt to the manufacturing area may be the reason for the high ^226^Ra concentration in this canal area. This is further confirmed by the soil sample taken at a distance of 100–150 m from the fertilizer factory, which contains a high level of radium (Fig. 3).

Moreover, the Ismailia Canal passes through different geologic formations, with sand and marl downstream and silt and mud upstream of the canal (El-Mathana et al. 2021). About 80% of Nile water from the Blue Nile and Atbara rivers originates from highland Ethiopia, mainly mafic and basalt rocks low in naturally occurring radioactive elements (Turekian and Wedepohl 1961). Therefore, the possible explanation for the high amount of ^226^Ra in the sediment is the impact of the Abu Za’baal Fertilizer & Chemical Company and the Aluminium Sulfate Company.

The precipitated radium from water into sediment may lead to the highest ^226^Ra concentration in sediment and the lowest ^226^Ra concentration in the water near the Aluminium Sulfate Company at the station (M8) during winter and at the station (M9) during summer. When highly saturated, radium tends to co-precipitate with sulfate to form radiobarite (RaSO_4_) rather than supersaturated carbonate minerals (Menzie et al. 2008; IAEA 2014). Over time, as radium with sulfate accumulates in easily carried fine-grained sediments, it mobilizes to locations with lower radioactive element concentrations (Elizabeth and Phillips 2001; Renock et al. 2016). At the same time, Ismailia Canal sediment was dominated by a small grain size (128 μm), possibly due to clay precipitation at the canal bottom (Youssef et al. 2004). Additionally, the Abu Za’baal region showed Oligocene basalt on the surface of the sediment, which is dominated by sands, gravel, and fractured basalt (Kandil et al. 2023).

The ^232^Th levels in sediment samples revealed minimal values, indicating that the industrial zone had little impact on the distribution of thorium. ^232^Th activity levels in sediment samples are substantially lower than those of Ra. This might be because the sediment contains Th as a very insoluble oxide, and the bioavailability of thorium is likewise very low, leading to the hypothesis of a common threat of Th-232 in industrial areas. Conversely, a relatively high concentration of ^40^K was determined in sediment samples in zone [B], more than in zones [A] and [C]. This might occur because one of the company’s commercial products (phosphoric calcium sulfate) has high potassium and calcium carbonate levels.

Furthermore, the limestone-based banks of the Nile Valley contribute to the Ca, Na, and HCO_3_ enrichment in the Ismailia Canal media (Ramadan et al. 2015). Anthropogenic activities cause variations in the concentrations of radionuclides, while others result from natural processes controlled by the mineralogy of the rock or soil. The results showed that the mean activity concentration of ^226^Ra,^232^Th, and ^40^K in sediment samples during winter is higher than during summer and lower than the worldwide value (UNSCEAR 2000).

#### Fertilizer samples

**Fig. 4:**
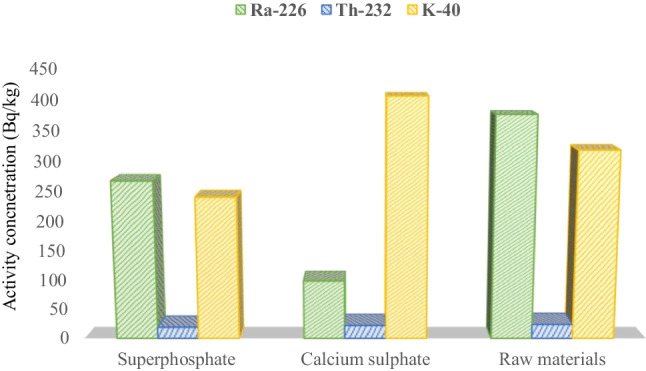
Comparison between the activity concentration of ^226^Ra, ^232^Th, and ^40^K for the commercial fertilizers (SSP and PCS) and the average value of raw materials

The radionuclide activity concentration in the commercial fertilizers and raw materials from Abu Za’baal Fertilizer & Chemical Company is illustrated in Table 3 and Fig. 4. Granulated single super phosphate (SSP) (17% P_2_O_5_) and phosphorus calcium sulfate (PCS) (0.5% P_2_O_5_, 6% calcium, and 13% sulfate) are two samples from the local market, in addition to two samples of raw materials. The activity concentrations of ^226^Ra, ^232^Th, and ^40^K for SSP were 268.11 ± 2.62, 19.48 ± 1.27, and 241.24 ± 4.28 Bq/kg, respectively. Likewise, for PCS, it was 99.64 ± 2.06, 22.51 ± 1.52, and 406.66 ± 5.49 Bq/kg, respectively. For the raw materials, the specific activity concentrations of ^226^Ra, ^232^Th, and ^40^K varied from 362.61 ± 2.53 to 390.23 ± 2.91, 23.56 ± 1.7 to 25.19 ± 1.12, and 310.21 ± 4.02 to 327.04 ± 4.62 Bq/kg, respectively. According to fertilizer investigation findings, raw material samples have higher ^226^Ra activity concentrations than commercial fertilizers (SSP and PCS). All fertilizer sample ^232^Th concentrations displayed shallow values. Additionally, a sample of phosphorus calcium sulfate fertilizer with K as its primary component exhibited a relatively high concentration of ^40^K. Furthermore, in a soil sample taken from a fertilizer company 100 to 150 m away, the activity of the radionuclides ^226^Ra, ^232^Th, and ^40^K was 73.2 ± 2.5, 18.51 ± 0.98, and 350.23 ± 4.98 Bq/kg, respectively. These findings are more significant than those of the control sediment sample and the global average value of ^226^Ra in soil samples (UNSCEAR 2000). The presence of waste products and the dust from raw materials that settle near the factory is likely responsible for this elevated concentration of ^226^Ra.

Table 4 compares ^226^Ra, ^232^Th, and ^40^K activity concentrations in water, sediment, and phosphate fertilizer samples from the study area to those previously investigated in different countries worldwide. The mean activity concentrations of ^226^Ra, ^232^Th, and ^40^K in the investigated water were consistent with those found in Lake Nasser’s freshwater (Imam et al. 2020), and it was lower than those found in the Ismailia Canal (Ramadan et al. 2015). Additionally, the ^226^Ra activity concentration was consistent with a water sample from the Nile River close to the Abu-Tartur fertilizer company (Mourad et al. 2009). On the other side, the activity concentration of ^40^K in this study was higher than those in the Ismailia Canal (Ramadan et al. 2015) and the water sample from the Nile River close to the Abu-Tartur phosphate fertilizer company (Mourad et al. 2009). Similarly, the mean activity concentration of ^226^Ra in the sediment aligns with those in the Ismailia Canal (Badawy et al. 2015), Lake Nasser (Imam et al. 2020), and the sediment sample from the River Nile next to the Abu-Tartur phosphate fertilizer company (Mourad et al. 2009). Furthermore, the activity concentration of ^232^Th agrees with the sediment sample from the River Nile next to the Abu-Tartur phosphate fertilizer company (Mourad et al. 2009) and Lake Nasser (Imam et al. 2020). The activity concentration of ^40^K agreed with Lake Nasser (Imam et al. 2020) and sediment samples from the Nile River close to the Abu-Tartur phosphate fertilizer company (Mourad et al. 2009) and was lower than the Ismailia Canal (Badawy et al. 2015).

**Table 4:** A comparison of the activity concentrations of ^226^Ra, ^232^Th, and ^40^K in water, sediment samples from the investigation area, and phosphate fertilizer samples from the Abu Za’baal Company with previous studies from different countries

Country	^226^Ra-series	^232^Th-series	^40^K	Note	Reference
Ismailia Canal	0.52 ± 0.07 28.61 ± 1.30	0.50 ± 0.8 14.51 ± 1.18	4.67 ± 0.48 261.41 ± 6.26	Water Sediment	Present study
Ismailia Canal	0.1–2.8	0.1–2.3	0.4–1.2	Water	Ramadan et al. (2015)
Ismailia Canal	24 ± 1.4	21 ± 1.1	323.8 ± 3.41	Sediment	Badawy et al. (2015)
Lake Nasser, Egypt	0.43 ± 0.08 26 ± 1.6	0.44 ± 0.1 19 ± 1.5	4.4 ± 0.93 255.6 ± 7.9	Freshwater Sediment	Imam et al. (2020)
A long the Nile River close to the Abu-Tartur phosphate fertilizer company, Egypt	0.06–1.3 15.4–33.8	0.02–0.16 10.4–19.3	0.14–0.6 128–281	Water Sediment	Mourad et al. (2009)
Abu Za’baal phosphate plant	268.11 ± 2.62	19.48 ± 1.27	241.24 ± 4.28	Granulated single superphosphate (SSP)	Present study
	99.64 ± 2.06	22.51 ± 1.52	406.66 ± 5.49	Phosphorus calcium sulfate (PCS)	
	376.42 ± 2.72	24.38 ± 1.41	318.63 ± 4.32	Raw materials	
Abu Za’baal phosphate plant, Egypt	301 ± 3.9	24 ± 1.4	3 ± 0.9	Superphosphate	Hussein (1994)
Abu-Tartur phosphate company, Egypt	778 ± 27 627 ± 24	12.4 ± 2.8 6.1 ± 2.4	51.6 ± 7.8 8.2 ± 2.2	Phosphate raw Superphosphate	Mourad et al. (2009)
Assuit fertilizer factory, Upper Egypt	445.50 ± 24.38	126.23 ± 6.31	192.22 ± 9.61	Superphosphate	El-Taher and Makhluf (2010)
Fertilizer samples from the Indian markets, India	527 ± 15	7 ± 0.2	87 ± 2.0	Superphosphate	Chauhan et al. (2013)
Brazil	375	100	871	Superphosphate	Saueia et al. (2005)
Saudi Arabia	55.2	8.86	553	Superphosphate	El-Taher and Abdelhalim (2013)
Bangladesh	143	-	292	Superphosphate	Alam et al. (1997)
Pakistan	221	49.7	556	Superphosphate	Khan et al. (1998

Table 4 compares the concentrations of ^226^Ra, ^232^Th, and ^40^K for phosphate fertilizer samples, specifically single super phosphate fertilizer, phosphorus calcium sulfate (PCS), and phosphate raw material worldwide. Regarding ^226^Ra, the single super phosphate fertilizer agrees with those found in the Abu Za’baal phosphate plant (Hussein 1994) and Pakistan (Khan et al. 1998). Whereas it was higher than that of Saudi Arabia (El-Taher and Abdelhalim 2013) and Bangladesh (Alam et al. 1997), it remained lower than the fertilizer samples from the Indian markets, India (Chauhan et al. 2013); Brazil (Saueia et al. 2005); Assuit fertilizer factory, Upper Egypt (El-Taher and Makhluf 2010); and Abu-Tartur phosphate company, Egypt (Mourad et al. 2009). The activity concentration of ^232^Th in phosphate fertilizer samples was higher than those found in Bangladesh (Alam et al. 1997); fertilizer samples from the Indian markets, India (Chauhan et al. 2013); Abu-Tartur phosphate company, Egypt (Mourad et al. 2009); and Saudi Arabia (El-Taher and Abdelhalim 2013), and it remained lower than Abu Za’baal phosphate plant (Hussein 1994); Pakistan (Khan et al. 1998); Assuit fertilizer factory, Upper Egypt (El-Taher and Makhluf 2010); and Brazil (Saueia et al. 2005). Moreover, the activity concentration of ^40^K aligns with the Assuit fertilizer factory, Upper Egypt (El-Taher and Makhluf 2010) and Bangladesh (Alam et al. 1997), yet remains lower than Brazil (Saueia et al. 2005), Pakistan (Khan et al. 1998), and Saudi Arabia (El-Taher and Abdelhalim 2013). Conversely, it is higher than levels found in the Abu Za’baal phosphate plant (Hussein 1994), Abu-Tartur phosphate company, Egypt (Mourad et al. 2009), and fertilizer samples from the Indian markets, India (Chauhan et al. 2013). Regarding raw materials, the activity concentration of ^226^Ra was lower than that of Abu-Tartur phosphate company, Egypt (Mourad et al. 2009), while ^232^Th and ^40^K were higher than those reported for Abu-Tartur phosphate company, Egypt (Mourad et al. 2009).

### Diatom community composition

A total of 84 species belonging to 12 orders (Achnanthales, Aulacoseirales, Bacillariales, Cymbellales, Eunotiales, Fragilariales, Naviculales, Rhabdonematales, Rhopalodiales, Surirellales, Thalassiophysales, and Thalassiosirales) and 23 genera were recorded over the investigated period (Rhabdonematales and Surirellales were recorded only in winter). Based on the number of species, the wealthiest two orders were Bacillariales (including 15 species across four genera, with the genus Nitzschia comprising 12 species) and Naviculales (including 15 species across three genera, with the genus Navicula comprising 13 species), followed by the order Cymbellales, encompassing ten species belonging to two genera *Cymbella* and *Gomphonema*.

**Fig. 5:**
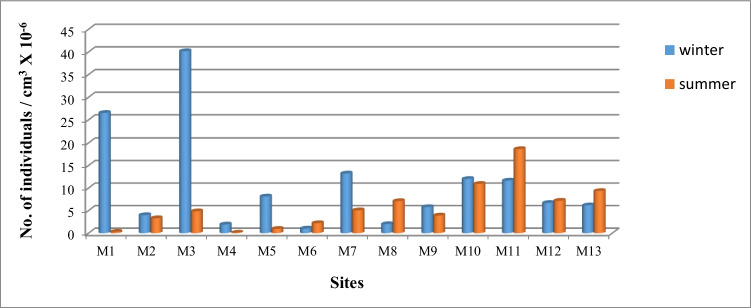
Spatial and temporal variations of diatom densities (individuals/cm^3^ × 10^−6^) at sites of Ismailia Canal during two seasons

The comparison did not display an evident seasonal variation (ANOVA, *P*-value = 0.15) in diatom abundance from winter to summer (Sherwood et al. 2000). This was evident in non-taxonomic attributes. However, the variability was evident among the sampling sites, potentially explaining changes in water trophic and ecological conditions. The spatial and temporal diatom distribution is represented in Fig. 5. The distribution of diatoms recorded in winter showed the highest density at sites (3) and (1), with abundances of 4 × 10^7^ cells/cm^3^ and 3 × 10^7^ cells/cm^3^, respectively. In summer, the highest density was recorded at site 11, followed by site 10 with 18.6 × 10^6^ cells/cm^3^ and 11 × 10^6^ cells/cm^3^, respectively. Diatom genera *Cyclotell*, *Fragillaria*, and *Aulacoseira* dominate the winter and summer peaks.

On the other hand, the lowest densities in winter were recorded at sites (6) (fertilizer Company), (4) (Petroleum Company drain), and (8) (Aluminium Sulfate Company drain), with 1 × 10^6^ cells/cm^3^ and 2 × 10^6^ cells/cm^3^, respectively. In summer, the least density was recorded at sites (4) and (1), with 0.12 × 10^6^ cells/cm^3^ and 0.32 × 10^6^ cells/cm^3^, respectively (Fig. 5). The spatial diatom densities were accompanied by the presence or absence of industrial activity. The highest densities were recorded at either zone [A] as in site one during winter (26.5 × 10^6^ cells/cm^3^) or [C] in site 10 (12.03 × 10^6^ cells/cm^3^ and 10.9 × 10^6^ cells/cm^3^ in winter and summer, respectively) and 11 (11.6 × 10^6^ cells/cm^3^ and 18.6 × 10^6^ cells/cm^3^ in winter and summer, respectively). Conversely, the lowest densities were recorded in zone [B], which included the industrial activities in the studied area of the canal, except at site 3 in winter, where the highest density of all parameters is still in the same range of zone [A]. The diatom community’s health, distribution, and the presence or absence of tolerant and sensitive species are significantly affected by the fertilizer company’s drain, leading to high phosphate and raw phosphate material waste. Besides the species of common occurrence (e.g., *Cyclotella ocellata*), samples collected at the fertilizer Company drain revealed a considerable density of tolerant and eutrophic species, *Nitzschia palea* (van Dam et al. 1994). *Nitzschia palea* is commonly associated with urban sites and areas polluted by residential and industrial effluents (Ndiritu et al. 2006). Generally, small species dominate stressful environments due to their fast metabolism (Falasco et al. 2009); however, large species are sensitive to stressful conditions. Additionally, some species may be more resistant to this radioactive stress than others.

### Influence of physicochemical and radioactive elements on diatom diversity, richness, and evenness

In terms of Shannon’s diversity index, the lowest diversity was recorded at sites (12) and (13), while the highest diversity occurred at sites (10) and (2) during winter and summer, respectively. Despite the high richness in diatom composition at each site, most sites were dominated by *Aulacoseira*, *Cyclotella*, and *Fragilaria* species without abnormalities. It is predicted that diversity, richness, and evenness will decline with increased human disturbance of habitats, leading to locations dominated by cosmopolitan species (*Aulacoseira*, *Cyclotella*, and *Fragilaria*), which are prevalent in most regions in the River Nile in Egypt. Richness during the winter ranged from 0.56 at the poorest site (2) to 1.64 at the highest site (10). In the summer, it ranged from 0.6 at the richest site (4) to 1.8 at the richest site (7). The highest abundance and dominance of *Aulacoseira granulata* at a site (1) in winter indicate the least evenness. Conversely, sites (12) and (13) represent the lowest evenness value in summer due to the high abundance of *Cyclotella ocellata* and *Fragilaria construens* species, constituting more than 85% of total diatom abundance. The correlation between diatom diversity, richness, evenness, and physico-chemical and radionuclides is presented in Table 5. Significant correlations (*P* < 0.05) were observed between diatom richness, diversity, evenness, and some physicochemical or radionuclide elements. Evenness was positively correlated with temperature in summer, EC, magnesium, R-226, and K-40. These conditions are not preferable for specific species, so the dominance was distributed fairly.

**Table 5:** Pearson correlation coefficient (*r*) between diatom (richness, evenness, diversity) and the physicochemical and radioactive elements measured in Ismailia Canal; Temp, temperature; E.C, electrical conductivity; BOD, biological oxygen demand; TP, total phosphorous. Ra-226, Th-232, and K-40 represent radionuclides activity during winter (w) and summer (S)

Diatom	Seasons	Temp °C	EC µS/cm	pH	BOD mg/l	NH^4+^ µg/l	SiO_2_ mg/l	SO_4_^−^ mg/l	TP µg/l	Na mg/l	K mg/l	Mg mg/l	Ca mg/l	Ra-226 Bq/kg	Th-232 Bq/kg	K-40 Bq/kg
Richness	**W**	0.098	− 0.446	0.077	0.196	**0.544**	− 0.115	− 0.325	− 0.131	− 0.462	0.134	** − 0.650**	0.330	− 0.377	0.111	− 0.312
	**S**	** − 0.550**	− 0.115	0.419	** − 0.580**	− 0.415	− 0.174	− 0.055	− 0.016	0.015	0.179	**0.549**	** − 0.740**	0.123	0.262	0.242
Evenness	**W**	− 0.013	**0.689**	− 0.210	− 0.237	** − 0.558**	0.160	0.361	0.027	0.439	− 0.057	**0.630**	− 0.322	**0.624**	0.117	**0.551**
	**S**	**0.745**	0.126	− 0.073	0.095	0.293	0.348	0.189	− 0.134	− 0.139	− 0.281	** − 0.533**	0.317	− 0.092	− 0.131	0.176
Diversity	**W**	0.117	0.256	0.073	− 0.024	0.118	− 0.024	− 0.096	− 0.233	− 0.025	0.072	− 0.070	0.058	0.396	0.264	0.388
	**S**	0.236	0.131	0.296	− 0.332	− 0.109	0.307	0.238	− 0.047	− 0.127	− 0.104	− 0.064	− 0.203	0.045	0.051	0.423

The bold emphasis represented the significance correlation

## Relationships between environmental variables, radionuclides, and biological community

### Environmental variables, major elements, and radionuclide analysis

**Fig. 6:**
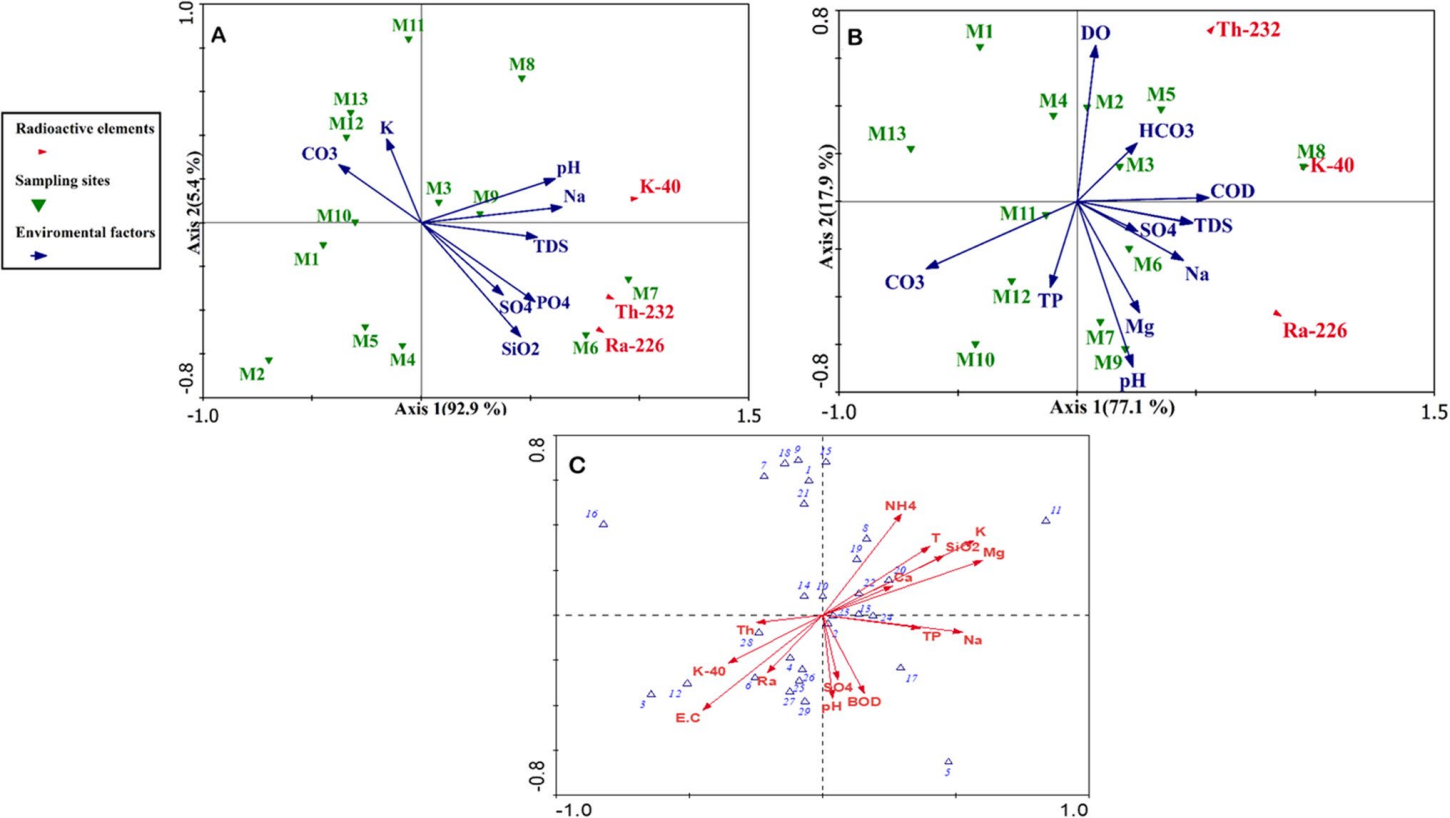
**A** Redundancy analysis (RDA) biplot of radioactive elements, environmental variables, nutrients, and major cations in water. **B** RDA of radioactive elements, environmental variables, nutrients, and major cations in sediment. **C** RDA of frequently recorded diatom taxa and water conditions (ammonium, NH_4_; carbonate, CO_3_; bicarbonate, HCO_3_; silicate, SiO_3_; sulfate, SO_4_ biological oxygen demand, BOD; pH; total phosphorus, TP; phosphate, PO_4_; electrical conductivity, E.C; Ra-226 series, Ra; Th-232 series, Th; and K-40 series, K) of Ismailia Canal

The findings of the multivariate redundancy analysis (RDA) for environmental factors, nutrient salts, significant elements, and radioactive elements in the water and sediment are shown in Fig. 6 A and B, respectively. In water, the RDA results for environmental variables, nutrient salts, and major elements with radionuclides for the first two axes accounted for 98.3% of the total variance, with the first two principal components (F1 = 92.9%, F2 = 5.4%). The analysis demonstrated a significant correlation between parameters (pH, Na, TDS, PO_4_, SO_4_, SiO_2_, K, and CO_3_) and radionuclides (Ra-226, Th-232, and K-40). A moderate positive correlation was observed between ^226^Ra and TDS, pH, PO_4_, Na, SiO_2,_ and SO_4_ (*r* > 0.5), indicating the precipitation of Ra-226 as soluble salts in the canal due to sulfate, as confirmed by Edsfeldt (2001). Correlations between Ra-226 and phosphorus and silicon dioxide at stations (M6) and (M7) further support the influence of the Aluminium Sulfate Company and Abu Za’baal Fertilizer and Chemical Companies. Statistical analysis revealed that the radioactivity elements ^226^Ra, ^232^Th, and ^40^K were linked with the high radioactive materials source. A negative correlation was also observed between Ra-226 and K (stable potassium) and CO_3_ (carbonate). Furthermore, a moderate positive correlation was noted between ^40^K and pH and CO_3._

Likewise, the RDA results for radionuclides and environmental variables, nutrient salts, and major elements in the sediment showed that axes 1 and 2 accounted for 77.1% and 17.9% of the total variation, respectively (Fig. 6B). The analysis indicated that the correlation between the parameters and elements behaves differently in sediment. Significant correlations were found between radionuclides and DO, HCO_3_, COD, TDS, SO_4_, Na, Mg, pH, TP, and CO_3_. This may reflect the relationship between the mineral dolomite (CaMg(CO_3_)_2_) found in the limestone and the presence of radionuclides (i.e., uranium and thorium series) in the limestone, consistent with previous findings (Kim 1995). A moderate positive correlation was also detected between Ra-226 and TDS, Na, and COD. These analyses show a statistically significant correlation between the physicochemical parameters (pH, TDS, and DO) and 226Ra, confirming that the behaviour of the radiation emitted by the different radionuclides depends on the overlying soil materials, the chelating agents, the physicochemical properties, and the type of rock (Belivermis et al. 2010). The correlation between COD and Ra-226 and K-40 suggests that more oxygen is needed to oxidize, indicating the possibility of additional oxidation (Camacho et al. 2012). Furthermore, there was a positive correlation between TP and K-40 and a weakly negative correlation with Ra-226 and Th-232.

### Taxonomic characteristics of diatoms with the tested variables

The multivariate redundancy analysis (RDA) of the ecological conditions showed that the non-impacted sites were significantly different from the impacted sites, indicating that the discharge of industrial effluents caused a modification of the physicochemical characteristics of the canal water, inducing enrichment of Ra-226, Th-232, and K-40, major cations (Ca^++^, Mg^++^, K^+^, and Na^+^), sulfate, total phosphorus, ammonium, and silicate. RDA analysis was also performed to test the effect of physical, chemical, and radionuclide elements on the diatom data (Fig. 6C). The analysis included species with a frequency of ≥ 30% (Table 6). Species-environment correlations were 0.9 and 0.8 with the first and second axes, respectively. The variance explained by all tested variables was 0.86 (*P* = 0.002), of which 0.21 was presented by Mg (*r* = 0.6, *P* = 0.004) and 0.15 was defined by the activity concentration of ^226^Ra, ^232^Th, and ^40^K (*r* =  − 0.2, 0.2, and 0.3, respectively). These findings confirmed that radioactivity threatens the diatom community composition (Millan et al. 2020). The accumulation of these radionuclides in the environment stresses the algae in different ways, including their effect on photosynthesis, chlorophyll production, and the functioning of enzymes (Banerjee et al. 2022).

**Table 6:** Seasonal average of relative abundance of most frequent (≥ 30%) attached diatom taxa identified from Ismailia Canal

RDA code	Species name	Currently accepted nomenclature	M1	M2	M3	M4	M5	M6	M7	M8	M9	M10	M11	M12	M13
1	*Achnanthidium minutissimum* (Kützing) Czarnecki		1.93	0	0.11	0.27	0.36	0	0.38	1.1	0	0.44	0.62	0.38	0.77
2	*Cocconeis placentula* Ehrenberg		0.95	0.33	0.17	4.68	0.43	1.16	0.3	0.39	0.14	0.25	0.66	0.4	0.89
3	*Cyclotella glomerata* H. Bachmann	Lindavia glomerata (H. Bachmann) Adesalu & Julius	8.36	10.9	13	12.2	7.52	9.48	14.3	15.9	10.4	6.83	12	5.22	3.69
4	*Cyclotella meneghiniana* Kützing	*Stephanocyclus meneghinianus* (Kützing) Kulikovskiy, Genkal & Kociolek	0.46	2.6	1.62	2.54	0.14	2.57	1.08	2.98	2.27	1.93	1.66	0.81	0.38
5	*Cyclotella ocellata* Pantocsek	*Pantocsekiella ocellata* (Pantocsek) K. T. Kiss & Ács	23.9	41.4	41.9	48	53.3	51.4	41.6	36.1	45.1	52.8	45.9	49.9	48.5
6	*Cymbella affinis* Kützing		0.04	0.17	0	0.4	0.27	0	0	0.08	0.19	0.09	0.05	0	0
7	*Cymbella microcephala* Grunow	*Encyonopsis microcephala* (Grunow) Krammer	0.9	0	0.14	0	0	0	0.08	0	0.19	0.05	0.08	0	0.18
8	*Epithemia sorex* Kützing		0.08	0.17	0.11	0	0	0	0.05	0.23	0.14	0	0	0	0.06
9	*Eunotia* sp.		0.95	0.17	0.36	0.13	0	0	0.05	0.08	0.47	0.2	0.46	0.24	0.18
10	*Fragilaria biceps* Ehrenberg		0	0.17	0.11	0.13	0	0	0	0.08	0.14	0	0.03	0	0
11	*Fragilaria construens* (Ehrenberg) Grunow	*Staurosira construens* Ehrenberg	14.2	11.7	10	3.48	16.2	17.2	17	21.1	14.7	15.2	23.2	36	38.3
12	*Fragilaria ulna* (Nitzsch) Lange-Bertalot	*Ulnaria ulna* (Nitzsch) Compère	2.11	2.38	1.13	3.74	1.99	0.91	2.59	0.78	1.62	1.48	4.66	0.41	0.33
13	*Fragilaria ulna* var. *acus* (Kützing) Lange-Bertalot	*Ulnaria acus* (Kützing) Aboal	0.04	0	0.17	0	0.36	0.37	0.22	0	0.33	0.09	0.27	0.16	0
14	*Gomphonema minutum* (C. Agardh) C. Agardh		0.04	0.17	0.11	0	0	0	0.05	0.63	0	0.09	0.14	0.16	0.09
15	*Gomphonema parvulum* (Kützing) Kützing		0.86	0	0	0	0	0	0	0.39	0	0.05	0	0.15	0.06
16	*Melosira granulata* (Ehrenberg) Ralfs	*Aulacoseira granulata* (Ehrenberg) Simonsen	40.9	23.1	23.8	17.8	15.9	7.95	10.2	13.6	20.4	8.5	8.66	3.79	3.04
17	*Melosira granulata* var. *angustissima* O. Müller	*Aulacoseira granulata* var. *angustissima* (O. Müller) Simonsen	0	0.83	0.11	0	0.43	1.82	0.51	0.47	1.18	0.96	0.37	0.15	0.18
18	*Navicula anglica* var. *subsalsa* (Grunow) Cleve	*Hippodonta subsalsa* (Grunow) Pomazkina & Radionova	0.95	0.33	0.14	0	0	0	0.05	0.08	0	0.14	0.06	0.08	0
19	*Navicula digitoradiata* (W. Gregory) Ralfs		0.08	0.17	0.03	0	0	0	0.05	0	0	0	0.09	0.08	0.06
20	*Navicula diluviana* Krasske	*Cymbellafalsa diluviana* (Krasske) Lange-Bertalot & Metzeltin	0	0	0.11	0	0	0	0.38	0	0.28	0	0	0.23	0
21	*Navicula pupula* Kützing	*Sellaphora pupula* (Kützing) Mereschkovsky	0.86	0.17	0	0	0	0.12	0.87	0	0	0	0	0	0
22	*Navicula salinarum* Grunow		0	0.17	0	0	0	0	0.31	0.08	0	0	0.06	0	0
23	*Navicula viridula* (Kützing) Ehrenberg		0.12	0	0	0.27	0	0	1.57	0	0.14	0.23	0.09	0	0.06
24	*Nitzschia dissipata* (Kützing) Rabenhorst		0	0	0.25	0	0	0.12	0	0.08	0	0.09	0.03	0.08	0
25	*Nitzschia frustulum* (Kützing) Grunow		0.08	1.1	0.41	0	0.87	0.37	0.6	0	0	2.75	0.06	0	0
26	*Nitzschia liebetruthii* Rabenhorst		0	1	0	0.4	0.27	0	2.67	0.08	0	0.79	0	0	0.06
27	*Nitzschia palea* (Kützing) W. Smith		0.25	0.72	1.37	2.41	0.83	3.85	1.18	0.08	1.38	0.43	0.06	0	0.3
28	*Nitzschia paleacea* (Grunow) Grunow		0.04	0.33	0.52	0.13	0.07	0.54	0.93	2.75	0	0	0	0	0
29	*Synedra tenera* W. Smith	*Fragilaria tenera* (W. Smith) Lange-Bertalot	0.04	0	0	0	0.14	0	0.17	0	0	0.37	0.05	0	0

Generally, under the effect of high-intensity radiation, the microalgae do not grow. However, if a specific type of microorganism has evolved under high-energy radiation, it can overcome the physiological stress in the harsh environment for growth and grow normally (Sukla et al. 2019). The impacted radioactive sites support a variety of algae species. Taxa such as *Cyclotella meneghiniana*, *Cymbella affinis*, *Nitzschia frustulum*, *Nitzschia liebetruthii*, *Nitzschia palea*, and *Synedra tenera* are associated with Ra-226 concentration. At the same time, *Cyclotella glomerata* and *Fragilaria ulna* are associated with naturally radioactive K-40, while *Nitzschia paleacea* is associated with a high Th-232 concentration. This association may involve the bioaccumulation of these radionuclides in diatom cells (Heidari et al. 2017) or their uptake for their metabolism. So, in our study, radionuclides do not eliminate diatom species, but some species show associations with radionuclide concentrations.

The major element (Na, K) positively correlated (*r* = 0.5) with the species’ first axis. As limiting factors affecting diatom community composition, total phosphorous (TP) and silicate (SiO_2_) seemed to explain some of the variance in the diatom community with a correlation matrix of 0.4. Species like *Navicula diluviana* and *Nitzschia dissipata* are associated with TP and SiO_2_ concentrations. *Navicula diluviana* has previously been classified as an indicator of TP (Van Dam et al. 1994; Belal 2012). NH_4_ is commonly considered one of the principal factors explaining the variation of diatom taxa and a growth-limiting factor (Enache and Prairie 2002). According to RDA (Fig. 8), NH_4_ can be considered a common factor in explaining the variability of the diatom communities with a correlation matrix of 0.3. *Epithemia sorex* and *Navicula digitoradiata* are correlated species, suggesting that these species can live in a wide range of ammonium concentrations (Belal 2012). While phosphorus and nitrogen are essential growth-promoting factors, their high concentrations, especially in agricultural areas, can affect diatom diversity, as seen in some affected sites. Meanwhile, the variance of the diatom community structure was slightly explained by pH and SO_4_, which did not significantly contribute to our understanding of the community structure of the diatoms found in the water of the Ismailia Canal. A group of commonly distributed species (*Achnanthidium minutissimum*, *Cymbella microcephala*, *Eunotia* sp., *Gomphonema parvulum*, *Navicula anglica* var. *subsalsa*, and *Navicula pupula*) was categorized, indicating a slight effect of most studied variables (Belal 2012).

## Water quality and radiological hazard indices

### Water quality index

**Fig. 7:**
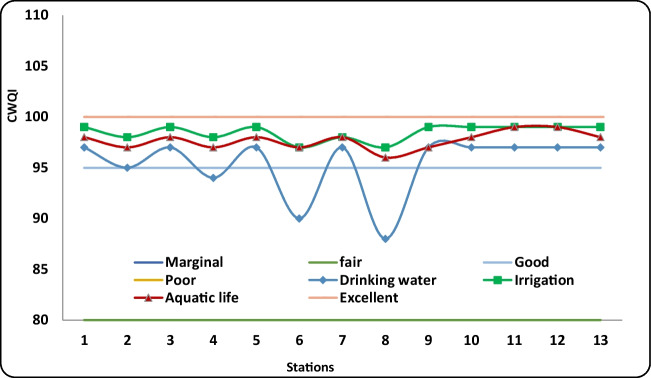
WQI of the Ismailia Canal at area under investigation for drinking, irrigation, and aquatic life utilizations

The values of WQI of Ismailia Canal are presented in Table 7 and illustrated in Fig. 7. Based on the WQI results, canal water can be considered an excellent source of drinking water, aquatic life, and irrigation with WQI 88–97, 97–99, and 96–99, respectively. Moreover, CWQI showed that Ismailia Canal water is suitable for drinking water, irrigation, and aquatic life use, consistent with El Sayed et al. (2023) findings.

**Table 7:** WQI and its categorizations of the Ismailia Canal at area under investigation for drinking, irrigation, and aquatic life utilizations

Stations	Drinking water		Irrigation		Aquatic life	
	CWQI	Rank	CWQI	Rank	CWQI	Rank
1	97	Excellent	99	Excellent	98	Excellent
2	95	Good	98	Excellent	97	Excellent
3	97	Excellent	99	Excellent	98	Excellent
4	94	Good	98	Excellent	97	Excellent
5	97	Excellent	99	Excellent	98	Excellent
6	90	Good	97	Excellent	97	Excellent
7	97	Excellent	98	Excellent	98	Excellent
8	88	Good	97	Excellent	96	Excellent
9	97	Excellent	99	Excellent	97	Excellent
10	97	Excellent	99	Excellent	98	Excellent
11	97	Excellent	99	Excellent	99	Excellent
12	97	Excellent	99	Excellent	99	Excellent
13	97	Excellent	99	Excellent	98	Excellent

### Radiation hazard indices

**Fig. 8:**
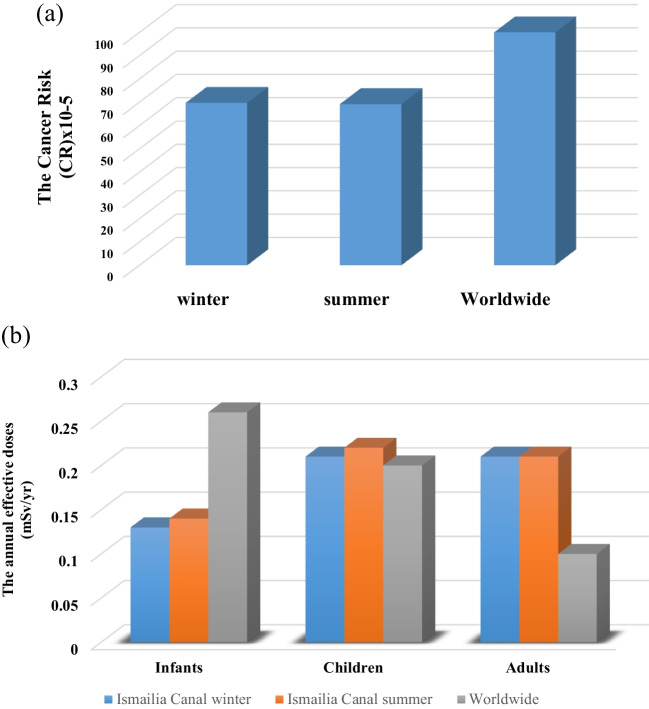
**a** The cancer risk associated due to the consumption of water samples and **b** the annual effective doses associated due to the consumption of water samples

Tables 8 and 9 present the most popular radiological hazard index in water and sediment through two seasons. The cancer risks caused by consuming radium isotopes found in water samples (^226^Ra and ^228^Ra) that ranged from 36.14 ± 2.78 × 10^−5^ to 137.45 ± 18.57 × 10^−5^ with an average value of 69.69 ± 12.97 × 10^−5^ in winter and from 31.91 ± 3.88 × 10^−5^ to 133.95 ± 10.25 × 10^−5^ with an average value of 69.16 ± 9.23 × 10^−5^ in summer, as represented in Table 8. The mean cancer risk values were within the acceptable range according to the USEPA (2000) standards, as represented in Fig. 8a. Effective doses were calculated for different age groups, including infants, children, and adults, considering the consumption of ^226^Ra, ^232^Th, and ^40^K in water samples during two seasons. The mean annual effective radiation dose for infants and children from drinking water in the study area is below the permissible effective dose limits set by the WHO in 2003. However, the mean annual effective doses of radiation for adults are higher than reference levels, as shown in Fig. 8b. The sites (M6) and (M7) near and downstream of the Abu Za’baal fertilizer company exhibited the highest cancer risk values and effective doses for various age groups in both seasons. In the same context, the mean absorbed dose rate of sediment during the winter and summer is 36.58 ± 1.90 and 29.55 ± 1.31 nGy/h, respectively, which is almost below the reference values provided in UNSCEAR (2000) Report 57 (18–93) nGy/h. In winter, the calculated values of Ra_eq_ ranged from 44.56 ± 3.29 to 144.74 ± 8.06 Bq/kg with an average of 74.72 ± 4.06 Bq/kg, while in summer, they varied from 29.01 ± 1.53 to 90.94 ± 3.51 with an average of 60.59 ± 2.79 Bq/kg. The annual effective dose for sediment samples varied from 0.026 ± 0.002 to 0.087 ± 0.005 mSv/year and between 0.018 ± 0.001 and 0.055 ± 0.002 mSv/year during winter and summer, respectively. Nevertheless, these values are lower than the worldwide limit (0.5 mSv/year) (UNSCEAR 2000) recommended. For sediment samples, the computed external hazard index fluctuated between 0.123 ± 0.009 and 0.402 ± 0.022 with an average of 0.207 ± 0.011 and from 0.081 ± 0.004 to 0.253 ± 0.010 with an average of 0.168 ± 0.008 during winter and summer, respectively. The mean external hazard is lower than the recommended maximum value, suggesting that the investigation area’s sediments are acceptable for use as building materials. The representative level index (*Iγ*) fluctuated between 0.331 ± 0.024 Bq/kg and 1.097 ± 0.058 Bq/kg and from 0.226 ± 0.011 Bq/kg to 0.690 ± 0.025 Bq/kg during winter and summer, respectively. For the reasons mentioned, the representative level index (*Iγ*) was higher than the worldwide reference value at (M8) during the winter. These results indicated that the mean *Iγ* values are less than the accepted recommended values (UNSCEAR 2000). Our findings revealed that the radium equivalent activities (Ra_eq_), the outdoor annual effective dose (*E*_eff_), the external hazard index (*H*_ex_), and the representative level index (*Iγ*) in consideration of the existence of natural radionuclides (^226^Ra, ^232^Th, and ^40^K) remain below the recommended maximum levels of UNSCEAR (2000) during both seasons.

**Table 8:** The annual effective doses and cancer risk associated with water consumption

Sample no	Cancer risk (CR) × 10^−5^		Total dose (mSv/year)					
	Winter	Summer	Winter			Summer		
			Infants	Children	Adults	Infants	Children	Adults
M1	44.95 ± 13.09	53.76 ± 10.31	0.09 ± 0.02	0.15 ± 0.03	0.12 ± 0.03	0.09 ± 0.01	0.15 ± 0.02	0.15 ± 0.02
M2	34.32 ± 12.51	51.87 ± 11.12	0.08 ± 0.02	0.14 ± 0.03	0.14 ± 0.03	0.09 ± 0.02	0.16 ± 0.03	0.15 ± 0.03
M3	72.14 ± 7.24	60.21 ± 10.46	0.12 ± 0.01	0.20 ± 0.01	0.17 ± 0.02	0.13 ± 0.02	0.2 ± 0.04	0.19 ± 0.03
M4	83.03 ± 17.62	87.47 ± 11.70	0.14 ± 0.02	0.21 ± 0.04	0.22 ± 0.04	0.13 ± 0.01	0.21 ± 0.02	0.22 ± 0.03
M5	49.48 ± 11.85	68.05 ± 7.61	0.12 ± 0.02	0.21 ± 0.04	0.22 ± 0.04	0.14 ± 0.01	0.23 ± 0.02	0.21 ± 0.02
M6	137.45 ± 18.57	133.95 ± 10.25	0.25 ± 0.02	0.42 ± 0.04	0.27 ± 0.04	0.28 ± 0.03	0.43 ± 0.05	0.42 ± 0.04
M7	128.16 ± 21.37	127.43 ± 15.21	0.24 ± 0.04	0.37 ± 0.08	0.51 ± 0.07	0.25 ± 0.02	0.39 ± 0.04	0.38 ± 0.04
M8	75.61 ± 11.78	96.83 ± 13.82	0.15 ± 0.02	0.23 ± 0.03	0.22 ± 0.03	0.18 ± 0.02	0.26 ± 0.03	0.27 ± 0.04
M9	96.02 ± 11.33	82.37 ± 9.00	0.16 ± 0.01	0.26 ± 0.02	0.21 ± 0.02	0.17 ± 0.02	0.26 ± 0.02	0.26 ± 0.02
M10	61.95 ± 13.74	35.87 ± 5.56	0.12 ± 0.02	0.19 ± 0.03	0.22 ± 0.03	0.09 ± 0.01	0.15 ± 0.02	0.13 ± 0.02
M11	45.79 ± 13.74	31.91 ± 3.88	0.1 ± 0.01	0.15 ± 0.03	0.16 ± 0.03	0.09 ± 0.01	0.14 ± 0.02	0.12 ± 0.02
M12	40.96 ± 13.01	33.51 ± 3.51	0.08 ± 0.01	0.13 ± 0.02	0.13 ± 0.03	0.08 ± 0.01	0.12 ± 0.02	0.11 ± 0.01
M13	36.14 ± 2.78	35.85 ± 7.61	0.08 ± 0.004	0.12 ± 0.01	0.12 ± 0.01	0.08 ± 0.01	0.12 ± 0.02	0.11 ± 0.02
Mean	69.69 ± 12.97	69.16 ± 9.23	0.13 ± 0.01	0.21 ± 0.02	0.21 ± 0.03	0.14 ± 0.02	0.22 ± 0.03	0.21 ± 0.03
Worldwide (WHO 2003; IAEA 1996)^a^ (USEPA 2000)^b^	10–100^b^		0.26^a^	0.2^a^	0.1^a^	0.26^a^	0.2^a^	0.1^a^

**Table 9:** The radiological hazard parameters in sediment samples during two seasons and phosphate fertilizer samples

Sample code	*D* (nGy/h)		Ra_eq_ (Bq/kg)		*E*_eff_ (mSv/year)		*H*_ex_		*Iγ* (Bq/kg)	
	Winter	Summer	Winter	Summer	Winter	Summer	Winter	Summer	Winter	Summer
M1	35.66 ± 1.67	14.66 ± 0.72	74.29 ± 3.59	29.01 ± 1.53	0.044 ± 0.002	0.018 ± 0.001	0.204 ± 0.010	0.081 ± 0.004	0.546 ± 0.026	0.226 ± 0.011
M2	28.59 ± 1.71	32.89 ± 1.71	58.16 ± 3.68	64.92 ± 3.57	0.035 ± 0.002	0.040 ± 0.002	0.162 ± 0.010	0.181 ± 0.010	0.442 ± 0.026	0.506 ± 0.026
M3	32.76 ± 1.97	35.52 ± 1.59	65.95 ± 4.20	73.28 ± 3.42	0.040 ± 0.002	0.044 ± 0.002	0.184 ± 0.012	0.203 ± 0.009	0.505 ± 0.030	0.547 ± 0.024
M4	32.83 ± 1.43	27.55 ± 1.72	65.64 ± 3.05	57.27 ± 3.67	0.040 ± 0.002	0.034 ± 0.002	0.183 ± 0.008	0.158 ± 0.010	0.506 ± 0.022	0.425 ± 0.026
M5	45.87 ± 3.22	31.65 ± 1.70	91.60 ± 6.69	64.30 ± 3.65	0.056 ± 0.004	0.039 ± 0.002	0.255 ± 0.018	0.179 ± 0.010	0.706 ± 0.049	0.489 ± 0.026
M6	40.98 ± 2.03	31.52 ± 1.89	83.65 ± 4.36	64.19 ± 4.06	0.050 ± 0.002	0.039 ± 0.002	0.232 ± 0.012	0.179 ± 0.011	0.632 ± 0.031	0.487 ± 0.029
M7	41.67 ± 1.19	32.33 ± 0.69	85.58 ± 2.53	66.70 ± 1.47	0.051 ± 0.001	0.040 ± 0.001	0.237 ± 0.007	0.185 ± 0.004	0.644 ± 0.018	0.500 ± 0.011
M8	71.06 ± 3.74	44.66 ± 1.62	144.74 ± 8.06	90.94 ± 3.51	0.087 ± 0.005	0.055 ± 0.002	0.402 ± 0.022	0.253 ± 0.010	1.097 ± 0.058	0.690 ± 0.025
M9	43.91 ± 1.34	41.44 ± 1.55	90.75 ± 2.78	84.67 ± 3.26	0.054 ± 0.002	0.051 ± 0.002	0.252 ± 0.008	0.235 ± 0.009	0.681 ± 0.021	0.641 ± 0.024
M10	23.84 ± 1.09	22.66 ± 1.11	49.70 ± 2.38	48.11 ± 2.38	0.029 ± 0.001	0.028 ± 0.001	0.137 ± 0.006	0.132 ± 0.006	0.368 ± 0.017	0.349 ± 0.017
M11	33.45 ± 1.80	21.50 ± 1.08	67.89 ± 3.84	44.77 ± 2.32	0.041 ± 0.002	0.026 ± 0.001	0.189 ± 0.011	0.124 ± 0.006	0.516 ± 0.028	0.331 ± 0.017
M12	23.29 ± 2.01	27.19 ± 0.80	48.84 ± 4.34	56.07 ± 1.72	0.029 ± 0.002	0.033 ± 0.001	0.135 ± 0.012	0.155 ± 0.005	0.359 ± 0.031	0.420 ± 0.012
M13	21.54 ± 1.53	20.54 ± 0.81	44.56 ± 3.29	43.48 ± 1.72	0.026 ± 0.002	0.025 ± 0.001	0.123 ± 0.009	0.119 ± 0.005	0.331 ± 0.024	0.316 ± 0.012
Mean (Canal)	36.58 ± 1.90	29.55 ± 1.31	74.72 ± 4.06	60.59 ± 2.79	0.04 ± 0.001	0.036 ± 0.002	0.207 ± 0.011	0.168 ± 0.008	0.564 ± 0.029	0.456 ± 0.020
Granulated single super phosphate (SSP)	137.75 ± 2.14		312.85 ± 4.7		0.17 ± 0.003		0.85 ± 0.01		2.14 ± 0.03	
Phosphorus calcium sulfate (PCS)	74.93 ± 2.12		160.29 ± 4.6		0.09 ± 0.003		0.44 ± 0.01		1.16 ± 0.03	
Raw materials	190.57 ± 2.28		433.59 ± 5.04		0.23 ± 0.003		1.18 ± 0.01		2.97 ± 0.04	
Worldwide (UNSCEAR 2000) soil	57		< 370		0.5		1		1

The radiological hazard index of phosphate fertilizer samples is represented in Table 9. The absorbed dose rates for fertilizer samples varied from 74.93 to 190.57 nGy/h, surpassing the worldwide values stated in UNSCEAR 2000. The Ra_eq_ content in fertilizer samples fluctuated between 160.29 and 433.59 Bq/kg, with higher values observed in raw materials. Except for the raw materials, the annual effective dose and the external hazard for the fertilizer samples were less than the worldwide range. The representative level index (*Iγ*) fluctuated between 1.16 and 2.97 Bq/kg, higher than the recommended maximum levels of UNSCEAR (2000).

## Conclusion

The Ismailia Canal serves as a crucial source of drinking water for society and contains valuable fisheries. The primary contributors to pollution in the Ismailia Canal are the discharge of untreated domestic sewage, agricultural run-off, and industrial effluent. This investigation aimed to assess the impact of industrial contaminants on the water quality, radioactivity levels, and biological composition and distribution (attached diatoms) in the Ismailia Canal. Furthermore, the pollutants’ environmental impact and health hazard indices in the Ismailia Canal are monitored. The anthropogenic disturbance plays a significant role in the biodiversity of aquatic biological communities, influencing species richness, evenness, ecological status, and fluctuation of radioactivity levels in the Ismailia Canal. This study showed that the BOD and COD values were only moderately clean, indicating higher levels of organic pollutants in surface waters. The WQI showed that the canal’s water quality was unstable and ranged from 88 (good) to 97 (excellent) for drinking water. WQI values for aquatic and irrigation water range from 96–99 and 97–92, meeting WHO and Egyptian guidelines. Furthermore, the activity concentrations of ^226^Ra, ^232^Th, and ^40^K in the water and sediment samples for both seasons remain within the permissible limits for natural radionuclides, except for some sites in the zone [B] (the industrial zone), which exceeded the guideline value. The fertilizer samples show higher ^226^Ra activity in the raw material samples than in the commercial fertilizers (SSP and PCS).

Pollution impacts the diversity of diatoms, altering the structure and abundance of diatom communities, as indicated by the analysis of the diatom data. The total number of diatoms/cm^3^ and species were significantly reduced at the pollution discharge sites, contrasting with the residential areas, contributing to the high diatom densities and species richness. These areas boost the amount of nutrients at the limit that support algal growth and flourishing. The ecological tolerance of some diatom species to radionuclide activity was demonstrated concerning the distribution and ecological preferences of diatom species, as revealed by the respective differences in richness, evenness, and Shannon’s diversity indices. The tolerance of microalgae assemblages to water quality parameters changed with industrial discharges, demonstrating the relevance of diatoms as biological indicators for this type of pollution. This association may involve the bioaccumulation of these radionuclides in diatom cells or their uptake into their metabolism. Consequently, these responses of some diatom species may allow further studies to use them in the phycoremediation process, which may be a promising opportunity in the water treatment in the Ismailia Canal. Multivariate redundancy analysis (RDA), explicitly examining ecological characteristics, radionuclides, and the diatom community, showed that the unaffected sites differed significantly from those affected.

The radiological hazard index of the water, including the risk of cancer, falls within the USEPA ranges. The effective radiation doses from water consumption for infants and children are below the permissible limits, though higher for adults. Regarding sediment, the radium equivalent activities (Ra_eq_), outdoor annual effective doses (*E*_eff_), external hazard index (*H*_ex_), and representative level index (*Iγ*) were listed below the advised maximum levels for both seasons. Consequently, to improve the water quality of the canal, the following technical recommendations are proposed: (1) To provide valuable data and information to communities and decision-makers, further in-depth long-term studies on physicochemical parameters, biological and radioactive levels of canal water, and eco-smart water treatment technologies should be carried out with the active participation of the local community; (2) promote waste reduction at source by enforcing existing laws and standards on pollution control and waste management; and (3) raise awareness among the people about the canal’s importance in the pollution of the Nile River.

## Data Availability

The datasets and materials used during the current study are available from the corresponding author on reasonable request.

## Appendix

Tables 10, 11, and 12.

**Table 10:** Quality control of radioactivity measurements comparison between measured values with certified reference values (Bq/kg)

Sample code	This analysis measured values (Bq/kg)			Certified reference values (Bq/kg)		
	^226^Ra	^232^Th	^40^K	^226^Ra	^232^Th	^40^K
IAEA-446	9.89 ± 0.5	11.1 ± 0.98	0.46 ± 0.3	9.34	10.5	0.44
IAEA-443	0.04 ± 0.002	0.05 ± 0.009	0.00188 ± 0.0002	0.039 ± 0.002	0.044 ± 0.002	0.00185 ± 0.0001
IAEA-410	10.4 ± 0.93	10.2 ± 1.3	-	10.1 ± 0.8	10.0 ± 1	-
IAEA-312	273.5	378.23	-	269	371.08	-

**Table 11:** CCME-WQI categories

Rating	CCME-WQI value	Remarks
Excellent	95–100	Water quality is protected with a virtual absence of threat or impairment; conditions very close to natural or pristine levels
Good	80–94	Water quality is protected with only a minor degree of threat or impairment; conditions rarely depart from natural or desirable levels
Fair	65–79	Water quality is usually protected but occasionally threatened or impaired; conditions sometimes depart from natural or desirable levels
Marginal	45–64	Water quality is frequently threatened or impaired; conditions often depart from natural or desirable levels
Poor	0.0–44	Water quality is almost always threatened or impaired; conditions usually depart from natural or desirable levels

**Table 12:** Radiation hazard indices and the health effect equations

Hazard indices	Equation	References
Absorbed dose rate in air (nGy/h)	*D* = 0.462 *C*_Ra_ + 0.621 *C*_Th_ + 0.0417 *C*_K_	UNSCEAR (2000)
Annual effective dose rates (mSv/year)	AED = *D* (nGy/h) × 8760 (h/year) × 0.7 (Sv/Gy) × 0.2 × 10^−6^	UNSCEAR (2000)
Radium equivalent activity (Bq/kg)	Ra_eq_ = *C*_Ra_ + 1.43 *C*_Th_ + 0.077 *C*_K_	Kurnaz et al. (2007)
External hazard index (*H*_ex_)	*H*_ex_ = (*C*_Ra_/370 + *C*_Th_/259 + *C*_K_ / 4810 ≤ 1	UNSCEAR (2000)
Representative level index (Bq/kg)	*Iγ* = $\frac{{C}_{{\text{Ra}}}}{150}+\frac{{C}_{{\text{Th}}}}{100}+\frac{{C}_{{\text{K}}}}{1500}$	Chad-Umoren and Umoh (2017)
*Canc*e*r risk*	(*CR*) = *MCL* × *RC* × *TWI*	EPA (1999); Abdellah and Diab (2012)
The effective radiation doses	DRW = AW × IRW × IDF	USEPA (1999)

*C*_*Ra*_, *C*_*Th*_, and *C*_*K*_ are the activity concentrations in becquerel per kilogram of ^226^Ra, ^232^Th, and ^40^K, respectively. The conversion coefficient from absorbed dose to effective dose is 0.7 Sv/Gy, and the outdoor occupancy factor is 0.2.

## Abbreviations

*CCME–WQI*: Canadian Council of Ministers of Environment Water Quality Index
*WAWQI*: Weighted Arithmetic Water Quality Index
*WQI*: Water Quality Index
*WHO*: World Health Organization
*FAO*: Food and Agriculture Organization
*EWQS*: Egyptian drinking water quality standards
*UNSCEAR*: United Nations. Scientific Committee on the Effects of Atomic Radiation
*IAEA*: International Atomic Energy Agency
